# ENO2 regulates CD4^+^ T cell pyroptosis via mitochondrial ROS to drive immunological non-response in HIV infection

**DOI:** 10.1128/mbio.01702-25

**Published:** 2025-09-25

**Authors:** Siyao Li, Heqiao Wang, Pan Wang, Tianling Yang, Jiaqi Li, Mei Liu, Yajing Fu, Yongjun Jiang, Zining Zhang, Hong Shang

**Affiliations:** 1 State Key Laboratory for Diagnosis and Treatment of Infectious Diseases, NHC Key Laboratory of AIDS Prevention and Treatment, National Clinical Research Center for Laboratory Medicine, The First Hospital of China Medical University, China Medical University 26488, Shenyang, China; 2 Key Laboratory of AIDS Immunology, Chinese Academy of Medical Sciences, Shenyang, China; 3 Key Laboratory of AIDS Immunology of Liaoning Province, Shenyang, China; Georgia Institute of Technology, Atlanta, Georgia, USA

**Keywords:** HIV, immune response, pyroptosis, ENO2, mitochondrial ROS

## Abstract

Approximately 10–40% of patients with acquired immune deficiency syndrome (AIDS) fail to restore the number of CD4^+^ T cells after antiretroviral therapy (ART). They are referred to as immunological non-responders (INRs) and have increased morbidity and mortality of AIDS and non-AIDS events. Pyroptosis is one of the key factors driving CD4^+^ T cell death in human immunodeficiency virus (HIV) infection, but its relationship with immune reconstitution and the underlying mechanisms is poorly understood. Through our *in vitro* experiments, we showed that the expression of enolase 2 (ENO2) decreased in INRs and inhibited ENO2-enhanced CD4^+^ T cell pyroptosis through the regulation of reactive oxygen species (ROS). Furthermore, we discovered that supplementation with phosphoenolpyruvate (PEP), the catalytic product of ENO2, could restore mitochondrial function and reduce the pyroptosis of CD4^+^ T cells. Our study clarified the ENO2-PEP-ROS-pyroptosis axis of CD4^+^ T cells in INRs and provided a novel therapeutic target for enhancing immune reconstitution in HIV infection.

**IMPORTANCE:**

The decrease in CD4^+^ T cell count is an important cause of poor immune reconstitution in HIV-infected patients. In this study, we analyzed the pyroptosis of T cells in HIV-infected patients with poor immune reestablishment and demonstrated how ENO2, a key enzyme in the glycolytic pathway, affects pyroptosis through mitochondrial ROS. Our results clarified the role of ENO2 in regulating CD4^+^ T cell pyroptosis in INRs and discussed its possible mechanism. This provides a new target for improving immune reconstitution and intervention in HIV infection.

## INTRODUCTION

The progressive decline of CD4^+^ T cells, an important feature of human immunodeficiency virus (HIV) infection, leads to immune dysfunction and death in patients with opportunistic infections and tumors (1–3). Although antiretroviral therapy (ART) can effectively inhibit viral replication and greatly reduce the morbidity and mortality of patients (2, 4), there are still 10–40% of patients with poor immune reconstitution after ART who are known as immunological non-responders (INRs) (5, 6). The incidence of acquired immune deficiency syndrome (AIDS) and non-AIDS events (tumor, cardiovascular disease, etc.) is high among INRs, which affects the quality of life of patients and increases the mortality rate (5, 7). Currently, the mechanism of immune non-response has not been clarified, and effective interventions are lacking. Recent studies have shown that reduced production and increased death of CD4^+^ T cells are critical factors leading to an immune non-response (5, 8). Early studies mostly attributed the death of CD4^+^ T cells in HIV infection to apoptosis (9). Increasing evidence shows that another inflammatory-mediated form of programmed death, pyroptosis, plays an important role in the loss of CD4^+^ T cells during HIV infection. Previous study has revealed that caspase-3-mediated apoptosis only leads to the death of a small number of CD4^+^ T cells effectively infected by HIV, whereas over 95% of CD4^+^ T cell death in resting lymphocytes is caused by caspase-1-mediated pyroptosis (10). When the virus has been effectively inhibited by antiviral therapy, apoptosis induced by direct viral infection is not the main cause of cell death, and the role of pyroptosis in the recovery of CD4^+^ T cells after ART cannot be ignored. The classic pathway of pyroptosis involves the NLRP3 inflammasome, which activates caspase-1, cleaves GSDMD, and causes it to release the N-terminal domain to form polypore channels, resulting in cell death and the release of proinflammatory factors such as IL-1β and IL-18 (11). In HIV infection, NLRP3 can be activated by abortive HIV infection, inflammatory stimulation, viral proteins, and so on. GSDMD can be cleaved by caspase-1, ultimately causing pyroptosis (10, 12). Currently, there is limited research on the role of pyroptosis in immune reconstitution after treatment of HIV infection. A study has shown that the increase in pyroptosis in mucosal-associated invariant T cells is related to an immune non-response (13). Other studies have detected the NLRP3 and caspase-1 genes in peripheral blood or LPS-stimulated peripheral blood mononuclear cells (PBMCs) and reported that NLRP3 and caspase-1 expressions are increased in INRs (14, 15). However, the relationship between CD4^+^ T cell pyroptosis and immune reconstitution in HIV infection and the underlying mechanisms remains poorly understood. Clarifying the key molecules and mechanisms involved in the regulation of CD4^+^ T cell pyroptosis in INRs is crucial for the intervention of immune reconstitution.

Studies focused on immune metabolism have demonstrated that changes in cellular metabolic homeostasis directly impact cell death and that a variety of metabolic signals, such as energy, lipids, and sugars, can trigger immune cell pyroptosis (16). Experiments in mice have shown that fumarate, an intermediate of citric acid cycle metabolism, can prevent macrophage pyroptosis and inflammatory cytokine secretion induced by succinylation of GSDMD (17). In an acidic environment, the level of α-ketoglutaric acid, an important metabolite of the TCA cycle, increases the level of reactive oxygen species (ROS), resulting in oxidative DR6 endocytosis, which then recruits and activates HeLa cell caspase-8 to cleave GSDMC, resulting in pyroptosis (18). ROS are common signals that activate the NLRP3 inflammasome, and sugars, lipids, and amino acids can induce mitochondrial ROS accumulation to activate NLRP3 and induce pyroptosis, which contributes to disease progression in various metabolic diseases (19). A study in mice has shown that elevated ROS levels can activate NLRP3 to cause hepatitis injury and induce pyroptosis of hepatocytes (20). NAC, a ROS scavenger, has been demonstrated to suppress NLRP3 inflammasome activation induced by H.pylori through scavenging intracellular ROS, consequently inhibiting caspase-1 activation and the subsequent release of IL-1β and IL-18 (21). In HIV infection, CD4^+^ T cells have abnormal metabolism of sugars, lipids, and amino acids (22); however, it remains unclear whether these metabolic abnormalities are related to ROS and trigger pyroptosis of CD4^+^ T cells in INR patients.

In our study, we identified the differences in T cell pyroptosis levels and the overall distribution characteristics of pyroptosis between IR and INR patients and identified enolase 2 (ENO2) as a key molecule regulating CD4^+^ T cell pyroptosis. We then explored the effects of ENO2 on mitochondrial ROS and oxidative phosphorylation by inhibiting enzyme activity and gene knockdown. This study clarifies the specific mechanism by which ENO2 regulates CD4^+^ T cell pyroptosis and identifies effective interventions to prevent pyroptosis. Our study provides new insights and directions for improving immune recovery and clinical outcomes in HIV-infected individuals after ART.

## MATERIALS AND METHODS

### Study subjects

In this study, 185 HIV-infected patients who had received ART were recruited, including 136 IR patients and 49 INR patients. The inclusion criteria for the ART group were as follows: mean treatment duration exceeding 2 years and viral load below 20 copies/mL at the time of testing. The inclusion criteria for the IR group were an increase in CD4^+^ T cell count by more than 20% from baseline at the time of testing. The inclusion criteria for the INR group were as follows: CD4^+^ T cell count < 350 cells/µL at the time of testing or an increase in the CD4^+^ T cell count of less than 20% after ART (Table S1). The subjects were all from the Care Clinic of the First Hospital of China Medical University.

### Bioinformatics data analysis

The transcriptome data of PD-1^+^ and PD-1^−^ CD4^+^ T cells were analyzed using the GEO data set public database GSE17606. The filter conditions for the differentially expressed genes (DEGs) were set as adj. *P* < 0.05 and fold change > 1.5. The public GEO data set GSE18233 was used to analyze the correlation between ENO2 and pyroptosis indicators caspase-1, NLRP3, IL-1β, and IL-18. In GSE18233, ENO2 was divided into ENO2 high and ENO2 low groups according to mRNA expression level, and the filtering conditions were set as adj. *P* < 0.05, of which 35 cases of ENO2^high^ group and 32 cases of ENO2^low^ group. The DAVID website (https://davidbioinformatics.nih.gov/) was applied to analyze DEGs in the KEGG signal pathway and GO analysis.

### Cell purification and culture

Peripheral blood was collected and processed using density gradient centrifugation to isolate PBMCs. CD3^+^ and CD4^+^ T lymphocytes were enriched by negative selection using the Human T cell and CD4^+^ T cell isolation kit (STEMCELL) according to the manufacturer’s protocol, depending on different assay requirements.

All primary PBMCs or lymphocytes were incubated at 37°C, 5% CO_2_ in RPMI 1640 medium (Hyclone) containing 10% fetal bovine serum (LONSERA) and 1% penicillin and streptomycin mixture (TBD Science).

### Flow cytometry

Whole blood was collected from the subjects and PBMCs were extracted. After negative selection of T cells, caspase-1-FITC (Bio-Rad), NLRP3-PE (Miltenyi), CD4-APC-Cy7 (BioLegend), and CD3-PE-Cy7 (BioLegend) staining was performed for pyroptosis. In addition, TIGIT-APC (BioLegend), PD-1-BV421 (BioLegend), CD38-BV510 (BioLegend), HLA-DR-PerCP-Cy5.5 (BioLegend), CD45RA-BV510 (BioLegend), and CCR7-BV421 (BioLegend) were used for exhaustion, activation, and differentiation surface marker staining. Live/dead cells were stained using the LIVE/DEAD Fixable Aqua Dead Cell Stain Kit (Invitrogen). MitoSOX Red Mitochondrial Superoxide Indicator (Thermo Fisher) was used to label mitochondrial ROS, MitoTracker Green FM (Thermo Fisher) was used to label mitochondrial mass, and MitoTracker Orange CM Ros (Thermo Fisher) was used to label mitochondrial membrane potential (MMP). PD-1^+^ and PD-1^−^ CD4^+^ T cells were sorted using the flow antibodies CD4-APC-Cy7 (BioLegend) and PD-1-FITC (BioLegend). The cells were divided into PD-1^+^ and PD-1^−^ CD4^+^ T cell subsets, and the two groups of cells were sorted by BD FACSAria IIu flow cytometry sorter. Peripheral blood T cells were detected and analyzed by BD Canto II flow cytometry and FlowJo v10.3 software.

### Real-time fluorescence quantitative PCR

Total cellular RNA was extracted using the RNeasy Plus Micro Kit (Qiagen), according to the manufacturer’s instructions. RNA reverse transcription was performed using Prime Script RT Reagent Kit (Takara), RT-qPCR was performed using TB Green Premix Ex Taq II (Takara), and relative mRNA quantification was performed by 2^−ΔΔCt^ method. β-Actin was used as an internal control (Table S2).

### Inhibition and knockdown of ENO2

ENO2 was inhibited by the addition of 10 µM ENO2 inhibitor ENOblock (MCE), and anti-CD3/CD28 Dynabeads (Gibco) were added and incubated at 37°C for 24 h for flow cytometry.

Negatively selected CD4^+^ T cells were transfected using a Human T Cell Nucleofector Kit (Lonza) and an electroporation apparatus (Lonza), following the manufacturer’s protocol. For knockdown assays, 100 pmol of either siENO2 (Thermo Fisher) or a negative control siRNA (Thermo Fisher) was electroporated into human primary CD4^+^ T cells. Transfections were performed using the U-014 electroporation program on the Nucleofector 2b device. Twenty-four hours following the completion of the electro-transfection of the cells, T cells were stimulated with anti-CD3/CD28 Dynabeads (Gibco) for 48 h at 37°C in 5% CO_₂_ in 96-well plates for subsequent treatments. In addition, the ENO2 knockdown efficiency was shown by RT-qPCR (Fig. S5B), and the result shows that ENO2 knockdown efficiency is greater than 50%.

### Detection of oxidative phosphorylation of CD4^+^ T cells

Whole blood was collected from the subjects, and CD4^+^ T cells were negatively selected and cultured in 48-well plates. In the experimental group, 10 µM ENO2 inhibitor ENOblock (MCE) and soluble CD3/CD28 tetramer stimulator (STEMCELL) were added, and in the control group, an equal volume of DMSO (Sigma) and soluble CD3/CD28 stimulator were added. After incubation at 37℃ for 24 h, mitochondrial stress was detected with a Seahorse XFp Cell Mito Stress Test Kit (Agilent) following the user’s manual. The cell culture plates were treated with a cell adhesion agent (Corning), after which the number of cells was adjusted and equilibrated at 37℃ for 20 min before detection with a Seahorse XF HS Mini (Agilent).

### Statistical analysis

All statistical analyses were performed by GraphPad Prism v10.1.0. For *in vitro* experiments, a parametric *t*-test or (ratio) paired *t*-test was applied when the data followed a Gaussian distribution. If the data were not Gaussian, the nonparametric Mann-Whitney test or Wilcoxon matched-pairs signed rank test was utilized. For the comparisons of data sets, RM one-way ANOVA was used to compare the data with a normal distribution, and the Friedman test was employed for data sets not with a normal distribution. Data were recorded as mean and standard deviation (SD); *P* values < 0.05 were considered statistically significant.

## RESULTS

### The pyroptosis of CD4^+^ T cells is increased in HIV-infected INRs, and PD-1 can distinguish pyroptotic from non-pyroptotic CD4^+^ T cells

**Fig 1:**
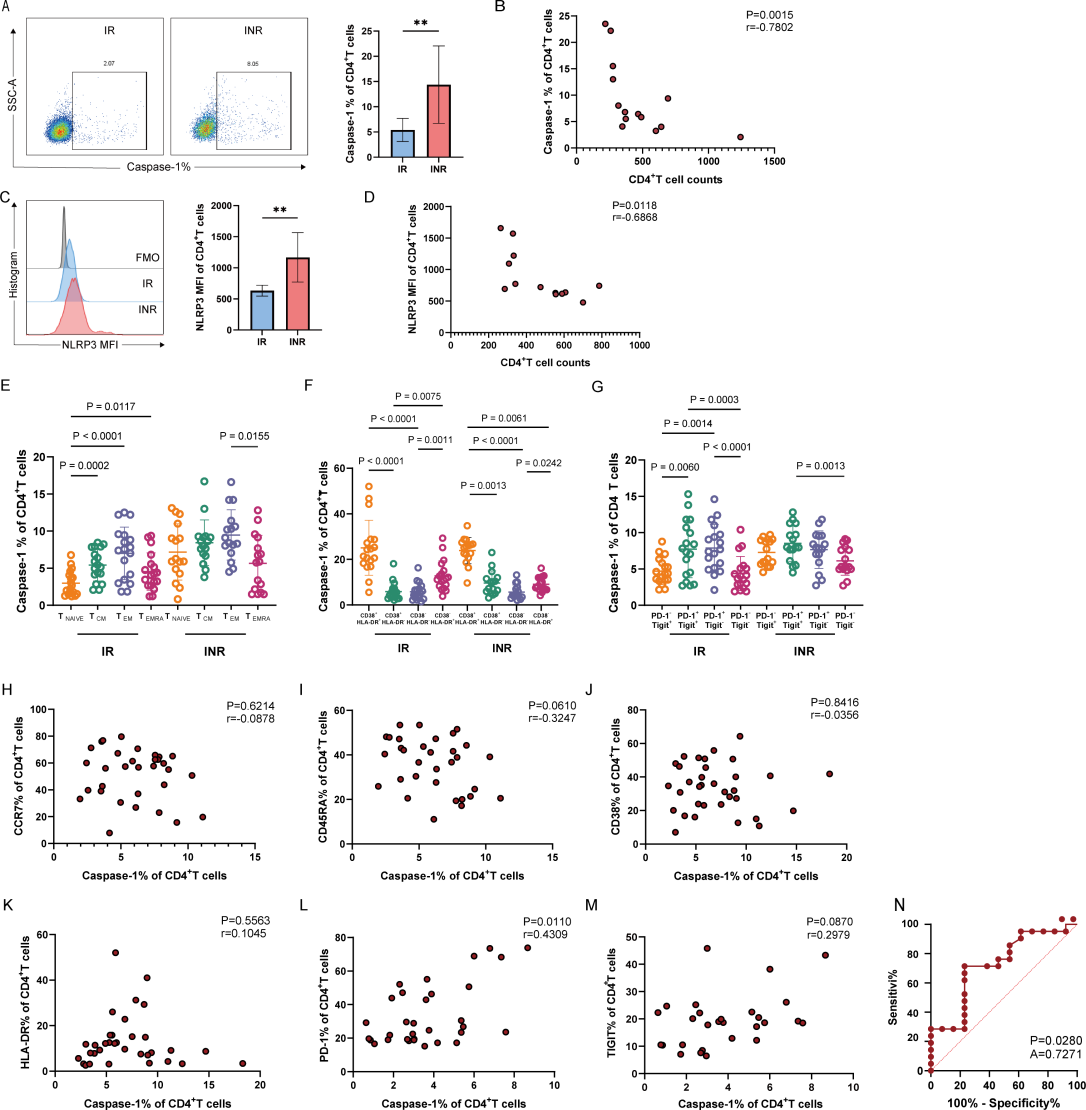
Pyroptosis characteristics of CD4^+^T cells in HIV patients after ART treatment. (**A**) Statistical graph of the difference in the percentage of caspase-1, an indicator of CD4^+^ T cell pyroptosis, between IR and INR patients on the right, and a typical flow graph of caspase-1 on the left (IR: *n* = 8, INR: *n* = 6). (**B**) Statistical graph of the correlation between the percentage of caspase-1 expression and the CD4^+^ T cells count (IR: *n* = 8, INR: *n* = 6). (**C**) Difference in NLRP3 inflammasome MFI of CD4^+^ T cells between IR and INR patients on the right, and typical flow graph of NLRP3 on the left (IR: *n* = 7, INR: *n* = 6). (**D**) Correlation between NLRP3 MFI of CD4^+^ T cells and CD4^+^ T cell counts (IR: *n* = 7, INR: *n* = 6). (**E**) Statistical graph of the proportion of CD4^+^ T cell pyroptosis indicator caspase-1 in each differentiated subset (IR: *n* = 18, INR: *n* = 16); T_NAÏVE_: CD45RA^+^CCR7^+^ naïve T cell subset; T_CM_: CD45RA^-^CCR7^+^ central memory T cell subset; T_EM_: CD45RA^-^CCR7^-^ effector memory T cell subset; T_EMRA_: CD45RA^+^CCR7^-^ terminally differentiated effector memory T cell subset. (**F**) Statistical graph of the proportion of CD4^+^ T cell pyroptosis indicator caspase-1 in each activated subset in IR and INR patients (IR: *n* = 18, INR: *n* = 16). (**G**) Statistical graph of the proportion of CD4^+^ T cell pyroptosis indicator caspase-1 in each exhausted subset in IR and INR patients (IR: *n* = 18, INR: *n* = 16). (**H–M**) Correlation analysis of CD4^+^ T cell pyroptosis indicator caspase-1 with differentiated indicators CCR7 and CD45RA, activated indicators CD38 and HLA-DR, exhausted indicators PD-1 and TIGIT (IR: *n* = 18, INR: *n* = 16). (**N**) ROC subjects working characteristic curve of PD-1 (*n* = 34). Data are analyzed by unpaired *t*-test in panels **A and C**. Spearman correlation analysis in panels **B, D,** and **H–M**. Friedman test in panels **E–G**. ROC curve in panel **N**. **P < 0.01.

To investigate the relationship between immune response and cellular pyroptosis after ART in HIV-infected patients, we examined the pyroptosis markers caspase-1 and NLRP3 in CD4^+^ and CD8^+^ T cells from HIV-infected patients. Our findings revealed that the level of caspase-1 in CD4^+^ T cells was significantly higher in INR patients than in IR patients (Fig. 1A) and that the level of caspase-1 in CD4^+^ T cells was significantly negatively correlated with CD4^+^ T cell count (Fig. 1B). In patients with INR, the NLRP3 expression level in CD4^+^ T cells was significantly elevated (Fig. 1C), the NLRP3 mean fluorescence intensity (MFI) of CD4^+^ T cells was significantly negatively correlated with the CD4^+^ T cell count (Fig. 1D), and similar results were observed for CD8^+^ T cells (Fig. S1A through D). We further detected pyroptosis markers, including IL-1β, IL-18, and GSDMD, of CD4^+^ T cells in IR and INR. We found that IL-1β released from CD4^+^ T cells in INR was higher than in IR by ELISA (Fig. S2A). Given that the released concentration of IL-18 was below the lower detection limit, we examined IL-18 mRNA levels and found that IL-18 mRNA of CD4^+^ T cells in INR was higher than in IR (Fig. S2B). We measured the GSDMD mRNA levels in CD4^+^ T cells of IR and INR and found that they were significantly elevated in INR patients (Fig. S2C). In addition, we assessed the levels of the apoptosis marker caspase-3 in CD4^+^ T cells in IR and INR, revealing significantly higher apoptosis in INR patients compared to IR patients (Fig. S3A). However, given the unclear relationship between pyroptosis and immune reconstitution, we prioritized investigating cellular pyroptosis as the focus of this study. Overall, our results indicate that the elevated level of T-cell pyroptosis in HIV-infected patients with poor immune response after ART may be one of the reasons why the number of CD4^+^ T cells in INR patients could not recover.

To further characterize the distribution of pyroptosis in CD4^+^ and CD8^+^ T cells from HIV-infected patients after ART, we analyzed the relationship between pyroptosis and the differentiation, activation, and exhaustion of these T cell subsets in HIV-infected individuals. We found that pyroptosis of CD4^+^ T cells in IR and INR patients was mainly distributed in the CD45RA^−^CCR7^−^ effector memory T cell subsets (T_EM_) (Fig. 1E), which was different from CD8^+^ T cells in INR (Fig. S1E). The pyroptosis of CD4^+^ and CD8^+^ T cells in IR and INR patients was mainly distributed in the CD38^+^ HLA-DR^+^ double-positive subsets (Fig. 1F; Fig. S1F). Notably, pyroptosis of CD4^+^ T cells in INR patients was mainly distributed in the PD-1^+^ TIGIT^+^ double-positive subset (Fig. 1G), whereas pyroptosis of CD4^+^ T cells in IR and the pyroptosis of CD8^+^ T cells in both groups were mainly distributed in the PD-1^+^ TIGIT^−^ subset (Fig. 1G; Fig. S1G). These findings suggest that CD4^+^ T cell pyroptosis in INR patients preferentially occurs in more differentiated, exhausted, and activated cell subsets. Additionally, we carried out statistical analyses on the caspase-1 percentages in these subsets across both patient cohorts. We identified significantly elevated caspase-1 levels in INR patients versus IR counterparts across multiple CD4^+^ T-cell subsets: in CD4^+^ T_Naïve_ cells, CD4^+^ T_CM_ cells, CD38^+^HLA-DR⁻ CD4^+^ T cells, PD-1⁻TIGIT^+^ CD4^+^ T cells, and PD-1⁻TIGIT⁻ CD4^+^ T cells. No statistically significant differences were observed in other cellular subsets between the cohorts (Fig. S2D through F). These findings provide clearer evidence that pyroptosis levels in CD4^+^ T cells are significantly elevated in INR.

Correlation analysis revealed a positive relationship between the expression of caspase-1 and PD-1 in CD4^+^ T cells (Fig. 1H thorugh M), which was different from CD8^+^ T cells (Fig. S1H through M). The heterogeneity of CD4^+^ and CD8^+^ T cells suggests the existence of different pyroptosis activation mechanisms. Furthermore, PD-1 expression significantly distinguished pyroptotic from non-pyroptotic CD4^+^ T cells (Fig. 1N), indicating that PD-1 may have an influence on the CD4^+^ T cell pyroptosis, and we aimed to explore PD-1 expression as a potential entry point to identify key molecules regulating CD4^+^ T cell pyroptosis.

### ENO2 and ENO3 may be the key metabolic regulatory genes involved in regulating CD4^+^ T cell pyroptosis

**Fig 2:**
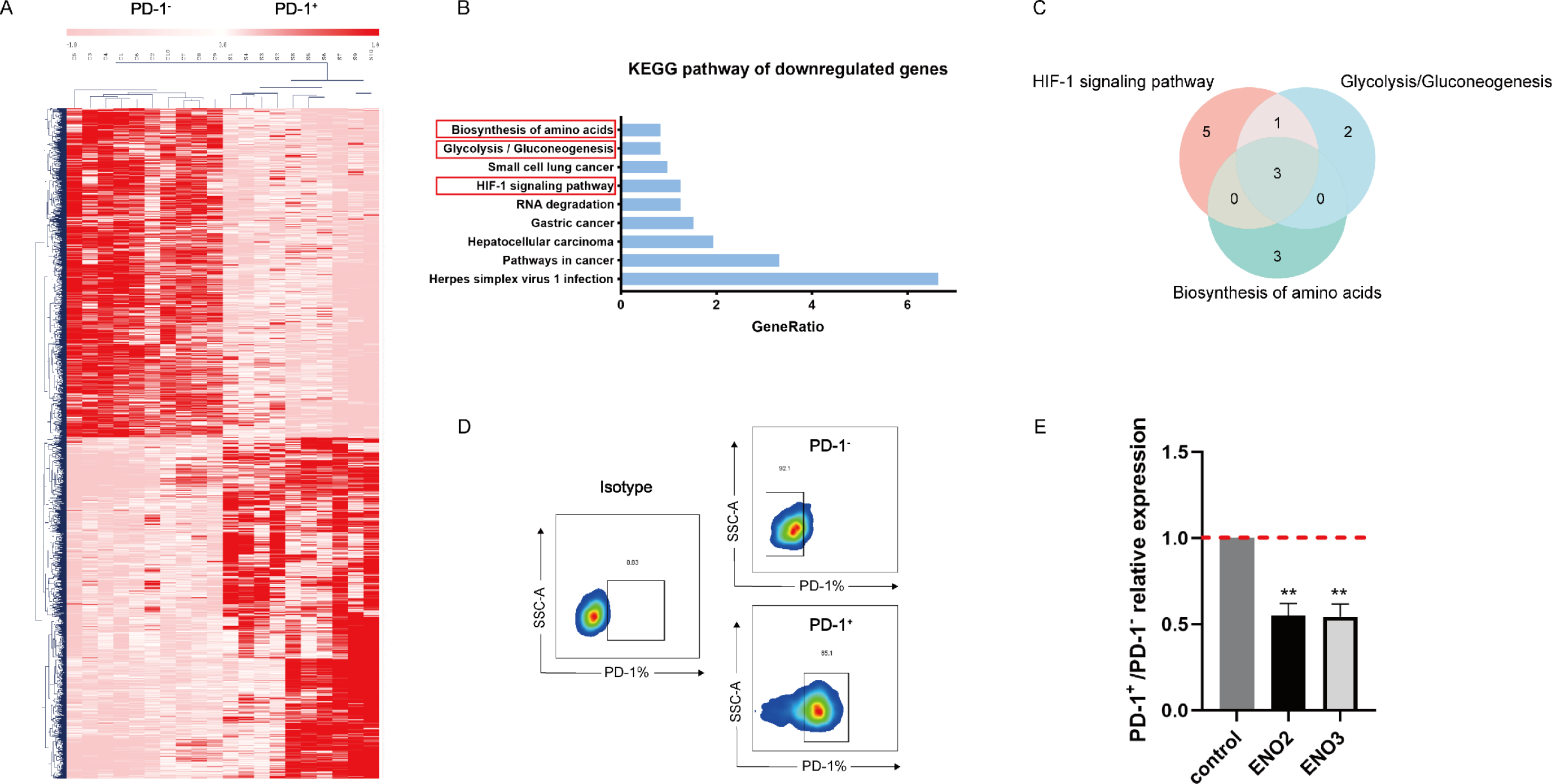
ENO2 and ENO3 may be the key metabolic regulatory genes in regulating cellular pyroptosis. (**A**) Analyze the difference in transcript levels between PD-1^+^ and PD-1^−^ CD4^+^ T cells by analyzing with GSE17606, and the filtering parameters were set to adj. *P* < 0.05, |fold change| > 1.5, and the differentially expressed genes were screened out, and unsupervised hierarchical clustering analysis and heatmap plotting were applied with the MeV software. (**B**) Analyze the downregulated genes for KEGG signaling pathway analysis using the DAVID website. (**C**) Venn diagrams of the intersections of genes involved in the amino acid biosynthesis, glycolysis, and HIF-1 signaling pathways. (**D**) Sorting purity of PD-1^+^ and PD-1^−^ CD4^+^ T cells by flow sorter. (**E**) Validation of the mRNA levels of PD-1^+^and PD-1^−^ CD4^+^ T cell glycolytic enzymes ENO2 and ENO3 (IR: *n* = 3). Data are analyzed by paired *t*-test in panel **E**. **P < 0.01.

To identify the key metabolic molecules that regulate the pyroptosis of CD4^+^ T cells, we further analyzed the GSE17606 data set. This data set showed differences in the transcriptional levels of PD-1^+^ and PD-1^−^ of CD4^+^ T cells from 20 HIV-infected individuals, with 10 PD-1^+^ and PD-1^−^ cases each. An unsupervised hierarchical clustering heatmap clearly distinguished the two groups of PD-1^+^ and PD-1^−^ CD4^+^ T cells (Fig. 2A). With adj. *P* < 0.05 and |fold change| > 1.5 as the thresholds, a total of 1,479 DEGs were screened, among which 727 were downregulated DEGs. KEGG pathway analysis of the downregulated DEGs revealed that they were enriched in nine signaling pathways: biosynthesis of amino acids, glycolysis/gluconeogenesis, small cell lung cancer, HIF-1 signaling pathway, RNA degradation, gastric cancer, hepatocellular carcinoma, pathways in cancer, and herpes simplex virus 1 infection (Fig. 2B). Among these, the biosynthesis of amino acids, glycolysis/gluconeogenesis, and the HIF-1 signaling pathway are strongly associated with cell metabolism. We further analyzed the genes involved in these three pathways (Fig. S4A through C) and found that the key enzymes of glycolysis, ENO2, ENO3, and ALDOC, might be the key metabolic regulatory genes (Fig. 2C).

As a rate-limiting enzyme in glycolysis, enolase can catalyze the conversion of 2-phospho-d-glyceric acid to phosphoenolpyruvate (PEP). In view of the key role of ENO in glycolysis (23–25), we selected ENO2 and ENO3 as key genes that may affect pyroptosis in CD4^+^ T cells for verification. To verify the above hypothesis, we sorted PD-1^+^ and PD-1^−^ CD4^+^ T cells (with a flow sorting purity of up to 85%; Fig. 2D) by flow sorter. Our results revealed significant reductions in the levels of the glycolysis enzymes ENO2 and ENO3 in PD-1^+^ CD4^+^ T cells compared with PD-1^−^ CD4^+^ T cells (Fig. 2E).

### ENO2 regulates CD4^+^ T cells pyroptosis in HIV-infected patients after ART

**Fig 3:**
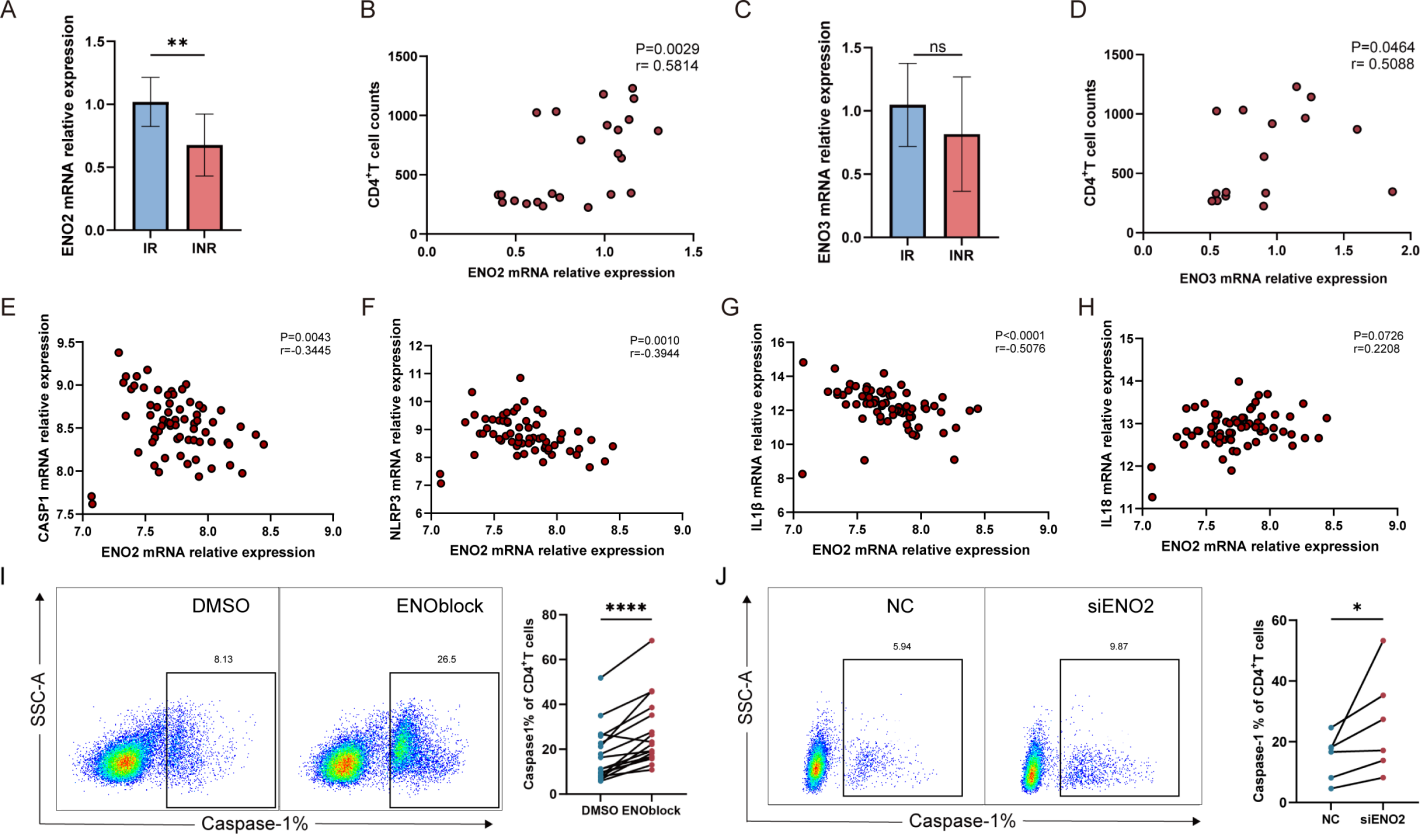
ENO2 was found to be a key metabolic regulatory molecule, which could regulate pyroptosis through screening and verification. (**A**) Difference in ENO2 mRNA expression levels between CD4^+^ T cells of IR and INR patients (IR: *n* = 12, INR: *n* = 12). (**B**) Correlation between ENO2 mRNA expression levels of CD4^+^ T cells and CD4^+^ T cell counts of ART patients (IR: *n* = 12, INR: *n* = 12). (**C**) Difference in ENO3 mRNA expression levels between CD4^+^ T cells of IR and INR patients (IR: *n* = 8, INR: *n* = 8). (**D**) Correlation between ENO3 mRNA expression levels of CD4^+^ T cells and CD4^+^ T cell counts of ART patients (IR: *n* = 8, INR: *n* = 8). (**E**) Correlation analysis between CD4^+^ T cell ENO2 and caspase-1 mRNA expression level in HIV-infected patients after ART (ART: *n* = 67). (**F**) Correlation analysis between CD4^+^ T cell ENO2 and NLRP3 mRNA expression level in HIV-infected patients after ART (ART: *n* = 67). (**G**) Correlation analysis between CD4^+^ T cell ENO2 and IL-1β mRNA expression level in HIV-infected patients after ART (ART: *n* = 67). (**H**) Correlation analysis between CD4^+^ T cell ENO2 and IL-18 mRNA expression level in HIV-infected patients after ART (ART: *n* = 67). (**I**) Detection of caspase-1 expression on CD4^+^ T cells 24 h after addition of the ENO2 inhibitor ENOblock in HIV-infected patients after ART, with typical flow graphs on the left and a statistical graph of percent change in caspase-1 levels on the right (*n* = 17). (**J**) Caspase-1 expression on CD4^+^ T cells was assayed after knockdown of ENO2 by siENO2 in HIV-infected patients after ART, with typical flow graphs on the left and a statistical graph of the percent change in caspase-1 levels on the right (*n* = 6). Data are analyzed by Mann-Whitney *U*-test in panels **A and C**. Spearman correlation analysis in panels **B, D and E–H**. Wilcoxon signed-rank test in panels **I and J**. ****P < 0.0001, **P < 0.01, and *P < 0.05. ART, HIV patients after antiretroviral therapy.

We assessed the expression levels of ENO2 and ENO3 in IR and INR patients by RT-qPCR and found that the expression levels of ENO2 mRNA were significantly lower in INR patients compared with IR patients (Fig. 3A), whereas the expression levels of ENO3 mRNA did not significantly differ between the two groups (Fig. 3C). Both the ENO2 and ENO3 mRNA expression levels were significantly positively correlated with the number of CD4^+^ T cells (Fig. 3B and D). To investigate whether ENO2 can regulate CD4^+^ T cell pyroptosis, we analyzed the relationship between ENO2 and CD4^+^ T cell pyroptosis in the GSE18233 data set. HIV-1 infected participants in this data set contributed samples under effective ART, and we selected cases from 67 successfully treated cases for analysis. The results showed that the ENO2 mRNA expression level was significantly negatively correlated with the pyroptosis indicator caspase-1, NLRP3, and IL-1β mRNA (Fig. 3E through G), but had no significant correlation with the IL-18 mRNA level (Fig. 3H).

In view of the close correlation between ENO2 and immune reconstitution and pyroptosis, we explored whether ENO2 can directly regulate CD4^+^ T cell pyroptosis. We added 10 µM ENOblock, an inhibitor of ENO2, to incubate CD4^+^ T cells for 24 h and detected the expression of caspase-1. The results showed that the level of CD4^+^ T cell pyroptosis was significantly elevated by the inhibition of ENO2 (Fig. 3I). Meanwhile, we used siRNA knockdown of ENO2 to detect the percentage of caspase-1 and death in CD4^+^ T cells and obtained the same result (Fig. 3J; Fig. S5A). Taken together, these results suggest that ENO2 is significantly reduced in INR patients and may play a regulatory role in the pyroptosis of CD4^+^ T cells in HIV-infected patients after ART.

### ENO2 regulates CD4^+^ T cell mitochondrial metabolism

**Fig 4:**
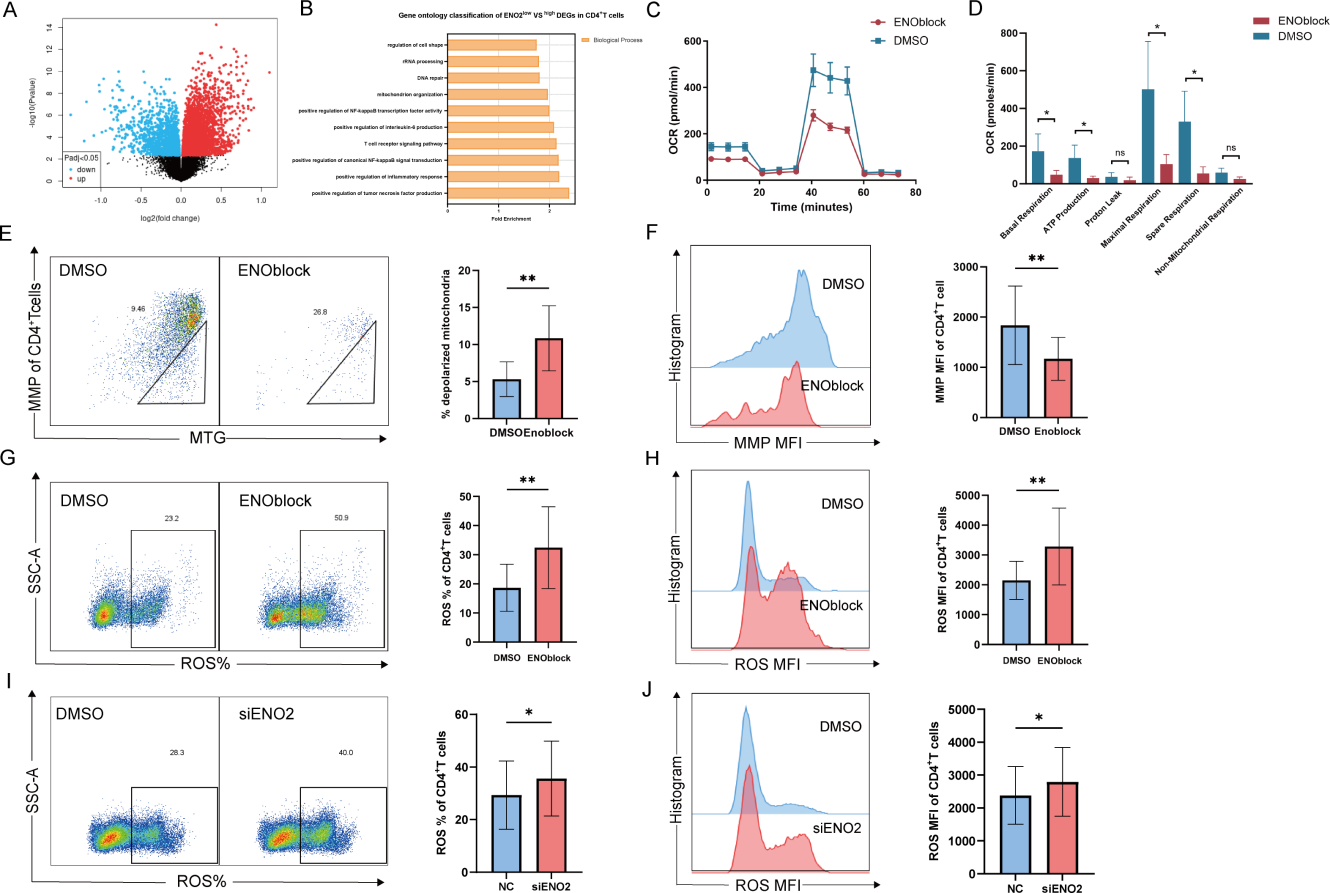
ENO2 regulates CD4^+^ T cell mitochondrial metabolism. (**A**) Volcano plot of ENO2^low^ and ENO2^high^ differentially expressed genes, adj. *P* < 0.05. (**B**) Gene ontology enrichment analysis of ENO2^low^ and ENO2^high^ differentially expressed genes. (**C**). Typical OCR graphs of negatively selected CD4^+^ T cells after the addition of 10 µM ENOblock and CD3/CD28 stimulation. (**D**) Statistical graph of key parameters after addition of 10 µM ENOblock. Basal respiration, ATP production, maximal respiration, and spare respiration were significantly reduced (*n* = 4). (**E and F**) Negative selection of CD4^+^ T cells from HIV-infected patients PBMC after ART, 10 µM ENOblock was added, and changes in the level of mitochondrial depolarization and mitochondrial membrane potential were detected after 24 h of incubation, with a typical flow graph on the left and a statistic of the percentage of depolarization and the membrane potential MFI statistic on the right (*n* = 9). (**G and H**) Negatively selected CD4^+^ T cells from PBMC of HIV-infected patients after ART, mitochondrial ROS were detected 24 h after the addition of 10 µM ENOblock, typical flow chart on the left, and percentage of ROS, MFI statistics on the right (IR: *n* = 9, INR: *n* = 1). (**I and J**) HIV-infected PBMC negatively selected CD4^+^ T cells after ART were added with 100 pm siENO2, a typical flow diagram was on the left, and the right was the mitochondrial ROS percentage statistic and mitochondrial ROS MFI statistic graph (*n* = 13). Data are analyzed by Ratio paired *t*-test in panel **D**. Wilcoxon signed-rank test in panels **E–J**. **P < 0.01 and *P < 0.05.

To investigate the mechanism by which ENO2 regulates CD4^+^ T cell pyroptosis, we analyzed the transcript levels of CD4^+^ T cells in 67 cases of HIV-infected patients after ART based on the GSE18233 data set. We categorized the data into two groups, ENO2^high^ and ENO2^low^, based on ENO2 mRNA expression levels (Fig. S6A). Taking adj. *P* < 0.05 as the threshold, a total of 3,850 DEGs were screened out (Fig. 4A). GO enrichment analysis revealed that the DEGs were mainly enriched in positive regulation of tumor necrosis factor production, positive regulation of the inflammatory response, positive regulation of canonical NF-kappa B signal transduction, the T cell receptor signaling pathway, positive regulation of interleukin-6 production, positive regulation of NF-kappa B transcription factor activity, mitochondrion organization, DNA repair, rRNA processing, and regulation of cell shape (Fig. 4B). Notably, Liu et al. recently reported that mitochondrial damage amplifies the downstream inflammatory response and cell pyroptosis in the early stage of pyroptosis (26). These findings underscore that mitochondrial damage precedes plasma membrane damage and may increase pyroptosis. Therefore, we hypothesized that ENO2 influences CD4^+^ T cell pyroptosis through pathways associated with mitochondrial metabolism.

To test this hypothesis, we negatively selected HIV-infected CD4^+^ T cells from ART-treated patients and co-incubated them with the ENO2 inhibitor ENOblock (10 µM) and CD3/CD28 stimulators for 24 h. Our results revealed a significant reduction in mitochondrial oxidative phosphorylation in CD4^+^ T cells (Fig. 4C), and that mitochondrial basal respiration, ATP production, maximal respiration, and spare respiration were significantly reduced after ENO2 inhibition (Fig. 4D). Moreover, inhibition of ENO2 significantly increased the level of depolarized mitochondria in CD4^+^ T cells (Fig. 4E), significantly decreased the MMP (Fig. 4F), and significantly increased the percentage and MFI of mitochondrial ROS and cytoplasmic ROS (Fig. 4G and H; Fig. S7A). In addition, the percentage of mitochondrial ROS and MFI was also significantly increased in CD4^+^ T cells after the knockdown of ENO2 (Fig. 4I and J). In summary, the above experimental results suggested that ENO2 regulated the CD4^+^ T cell mitochondrial metabolism and thus may affect pyroptosis.

### PEP supplementation reduces mitochondrial ROS and pyroptosis in CD4^+^ T cells

**Fig 5:**
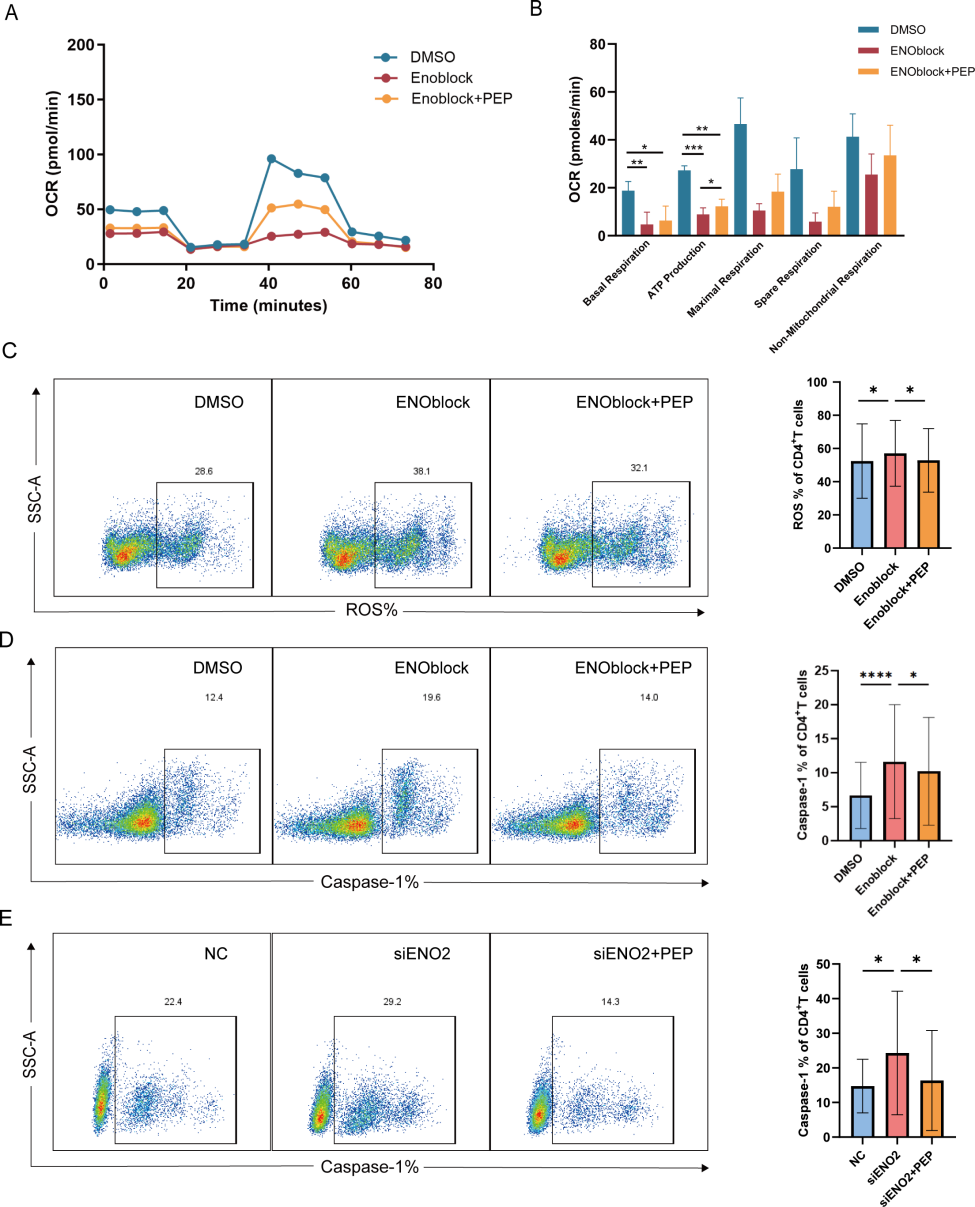
Phosphoenolpyruvate supplementation was found to partially restore mitochondrial oxidative phosphorylation, reduce mitochondrial ROS level, and reduce pyroptosis in CD4^+^ T cells. (**A and B**) Negatively selected HIV-infected CD4^+^ T cells after ART were incubated with 10 µM ENOblock with or without 10 µM PEP for 24 h to detect mitochondrial respiratory capacity OCR (IR: *n* = 3, INR: *n* = 1). (**C and D**) Negatively selected ART-treated HIV-infected CD4^+^ T cells, 10 µM ENOblock was added, incubated with or without 10 µM PEP for 24 h to detect mitochondrial ROS (IR: *n* = 10, INR: *n* = 3) and levels of pyroptosis (IR: *n* = 20), typical flow charts are shown on the left, and the statistical graph of the percentage is shown on the right. (**E**) Caspase-1 expression on CD4^+^ T cells was assayed after knockdown of ENO2 in HIV-infected patients after ART with siENO2, accompanied by exogenous supplementation of PEP, with typical flow charts on the left and a statistical plot of the percent change in caspase-1 levels on the right (*n* = 7). Data are analyzed by RM one-way ANOVA test in panel **B**; Friedman test in panels **C–E**. ****P < 0.0001, ***P < 0.001, **P < 0.01, and *P < 0.05.

ENO2 is a key enzyme in the glycolytic pathway, and its catalytic product is PEP, which may function as an antioxidant and help reduce mitochondrial ROS (27, 28). To address the question of how ENO2 influences pyroptosis by affecting mitochondrial metabolism, we assumed that PEP is a key factor in the regulation of mitochondrial ROS and pyroptosis by ENO2. We hypothesized that the reduced expression level of ENO2 in CD4^+^ T cells from INR patients might lead to decreased production of PEP and reduce the antioxidant capacity of the cells, leading to the accumulation of mitochondrial ROS, impairing mitochondrial ATP synthesis, and ultimately contributing to CD4^+^ T cell pyroptosis. Next, we examined whether the inhibition of ENO2 induced reduction in the oxidative phosphorylation level of CD4^+^ T cells could be restored by supplementation with 10 µM PEP. Our results showed that the oxidative phosphorylation level decreased after inhibition of ENO2, and the supplemental PEP could partially restore the oxidative phosphorylation level and significantly restore the ability to produce ATP (Fig. 5A and B).

Next, we measured the changes in mitochondrial ROS and pyroptosis levels following ENO2 inhibition by ENO inhibitor ENOblock and PEP supplementation. The results demonstrated a significant increase in mitochondrial ROS levels and pyroptosis in CD4^+^ T cells after ENO2 inhibition. Importantly, supplementation with PEP significantly reduced the mitochondrial ROS levels (Fig. 5C) and pyroptosis levels of CD4^+^ T cells (Fig. 5D). Given that the ENO inhibitor can not only inhibit ENO2 but also inhibit ENO1 and ENO3, we used siRNA to knock down ENO2. We also found that the pyroptosis of CD4^+^ T cells increased after ENO2 knockdown, but it was significantly reduced after supplementing with PEP (Fig. 5E). In addition, we recruited untreated HIV-infected individuals and isolated CD4^+^ T cells from these patients, who had a higher proportion of CD4^+^ T cells infected with HIV. The isolated CD4^+^ T cells were treated with ART drug azidothymidine (AZT) for 24 h, then with PEP for 24 h, and the percentage of caspase-1 was detected. Our findings revealed that PEP reversed HIV-mediated pyroptosis in CD4^+^ T cells (Fig. S8A), suggesting PEP may harbor therapeutic potential for restoring immune function in HIV-infected cells.

We further investigated the effects of PEP on activation, exhaustion, differentiation phenotypes, and apoptosis of CD4^+^ T cells. We observed that ENO2 inhibitor treatment significantly reduced the expression of activation markers (CD38 and HLA-DR) and exhaustion markers (PD-1 and TIGIT) in CD4^+^ T cells. This is likely attributable to ENO2’s essential role in glycolysis; loss of ENO2 activity impairs metabolic initiation of CD4^+^ T cell activation, thereby preventing subsequent exhaustion marker expression. Following ENO2 inhibition, we observed an increased proportion of T_EM_ and T_EMRA_ subsets, which may result from metabolic reprogramming toward alternative energy pathways upon glycolytic suppression. We found that PEP supplementation failed to fully reverse the effects of ENO2 inhibition on these phenotypes (Fig. S9A through H). This suggests that ENO2 may influence the activation, exhaustion, and differentiation of CD4^+^ T cells through alternative pathways in addition to the PEP metabolic pathway. Furthermore, we observed that inhibiting ENO2 significantly increased the level of the apoptotic marker caspase-3. Administration of PEP slightly reduced caspase-3 level in CD4^+^ T cells, suggesting that PEP may also attenuate CD4^+^ T-cell apoptosis by reducing ROS (Fig. S3B).

In conclusion, our results suggest that supplementation with exogenous PEP could partially restore the oxidative phosphorylation level of CD4^+^ T cells, the mitochondrial ROS level of CD4^+^ T cells, and the pyroptosis level of CD4^+^ T cells.

## DISCUSSION

**Fig 6:**
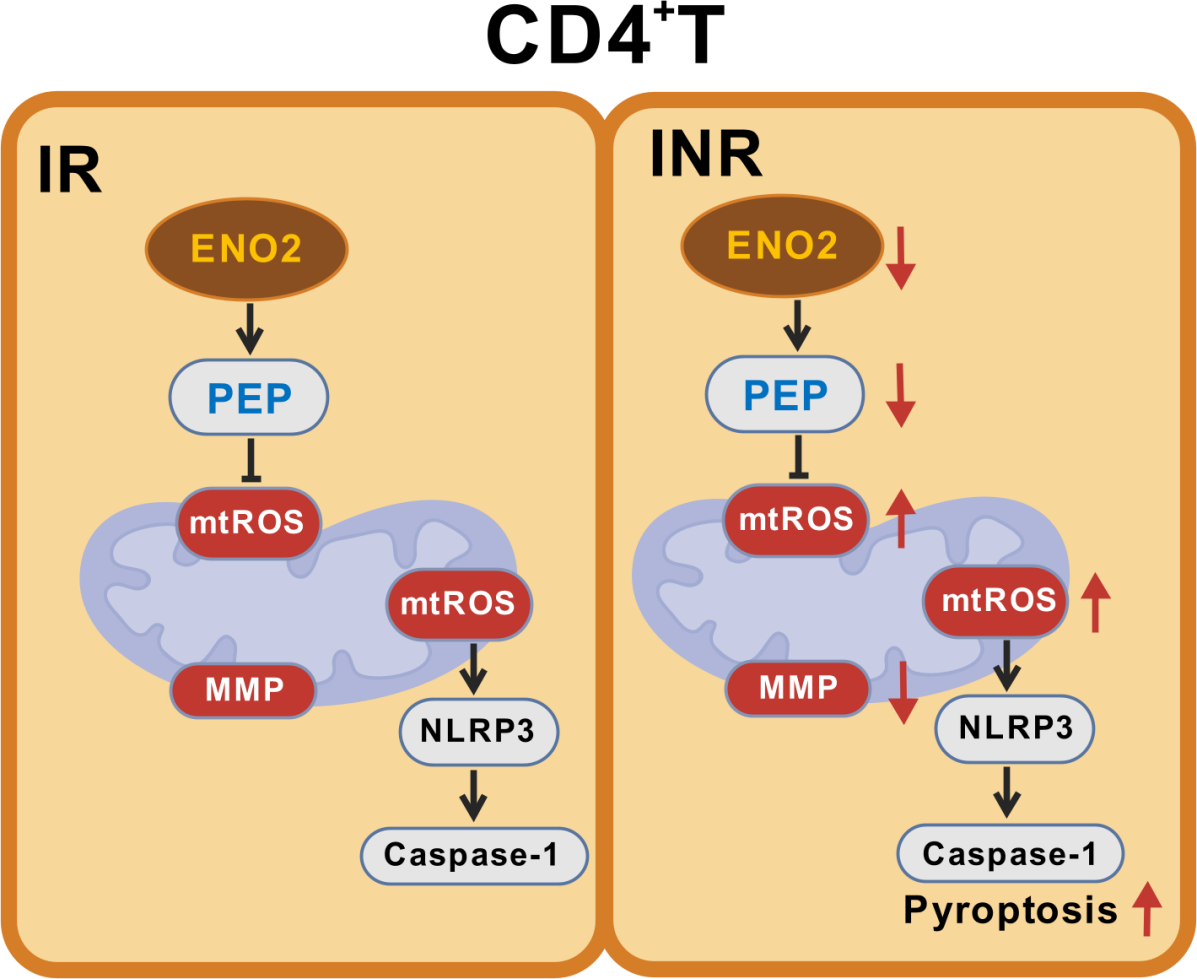
Schematic diagram of the effect of ENO2 on pyroptosis of CD4^+^ T cells by regulating mitochondrial ROS.

While the widespread use of ART has significantly improved the outcome of HIV infection, the number of CD4^+^ T cells in INRs cannot be effectively restored (29–31). Pyroptosis is a well-defined and important form of programmed cell death. In our study, we discovered increased protein levels of caspase-1 and NLRP3 in the CD4^+^ T cells of INR patients, suggesting that pyroptosis may be one of the reasons for poor immune recovery in INR patients. In addition, we also observed elevated apoptosis levels of CD4^+^ T cells in INR. Given the unclear mechanistic link between pyroptosis and immune reconstitution, we further focused our investigation on elucidating the pyroptosis mechanisms in CD4^+^ T cells. We found that the expression of the key metabolic molecule ENO2 was significantly reduced in the CD4^+^ T cells of INR patients and was positively correlated with disease progression. Inhibition of ENO2 impaired the mitochondrial function of CD4^+^ T cells, leading to the accumulation of mitochondrial ROS and subsequent pyroptosis (Fig. 6). Notably, treatment with exogenous PEP reversed the negative effects of ENO2 inhibition on mitochondrial ROS levels and pyroptosis in CD4^+^ T cells.

ENO2 is a crucial glycolytic enzyme that catalyzes the only dehydration step in the glycolytic pathway, converting PEP from phosphoglycerate (32). ENO has three isoenzymes: ENO1, ENO2, and ENO3. Although all three isozymes may potentially impact pyroptosis, our bioinformatic analysis identified ENO2 and ENO3 (rather than ENO1) as the key metabolic genes in CD4^+^ T cells. Critically, ENO2 showed more pronounced differential expression between the IR and INR compared to ENO3. Thus, despite certain limitations, our study focuses on investigating the role of ENO2 in regulating CD4^+^ T cell pyroptosis. Studies have reported that the expression of ENO2 is closely associated with the prognosis and treatment of various types of cancer (33–37). Our study found that ENO2 may regulate the pyroptosis mechanism of CD4^+^ T cells through the modulation of mitochondrial metabolism. Specifically, the inhibition of ENO2 resulted in decreased levels of oxidative phosphorylation in CD4^+^ T cells, along with reductions in basal respiration, ATP production, maximal respiration, and spare respiratory capacity. Furthermore, the inhibition of ENO2 led to a decrease in the membrane potential and an increase in the depolarization levels in CD4^+^ T cells. The MMP supports ATP production during oxidative phosphorylation, and a lower membrane potential may cause impairment of T cell mitochondrial function, increasing the level of depolarized mitochondria (38, 39), and mitochondrial damage plays a critical role in pyroptosis (26). In addition, we further discovered that the inhibition of ENO2 led to a notable increase in mitochondrial and cytoplasmic ROS. Excessive ROS-induced long-term oxidative stress can induce cellular senescence, malignant transformation, or death (40). Previous studies have demonstrated that imbalanced mitochondrial homeostasis can promote NLRP3 inflammasome activation (41), with ROS serving as common activators of NLRP3 inflammasome activation (19, 42–46). Simultaneously, there exists a close relationship between ROS and apoptosis. ROS is a crucial inducing factor for apoptosis, and ENO2 may also regulate cell apoptosis by influencing ROS. Based on the above, our current data suggest that the inhibition of ENO2 impairs the mitochondrial function of CD4^+^ T cells and leads to the accumulation of mitochondrial ROS, which may trigger NLRP3 inflammasome activation and lead to CD4^+^ T cell pyroptosis in INRs.

Our research found that inhibiting ENO2 while supplementing PEP can partially restore the oxidative phosphorylation level of CD4^+^ T cells, reduce mitochondrial ROS levels, and decrease pyroptosis. As the catalytic product of ENO2, PEP can be catalyzed by pyruvate kinase to produce pyruvate. Studies have shown that PEP supports T-cell function through two mechanisms: promoting cytokine production via the calcium ion signaling pathway (27), and serving as a substrate for generating pyruvate (47). Pyruvate can enter the tricarboxylic acid cycle for oxidative phosphorylation to generate ATP, providing energy for the cells. A study has indicated that CD8^+^ T cells infiltrating tumors exhibit decreased ENO enzyme activity, metabolic defects in glycolysis and oxidative phosphorylation, and exhaustion of function; however, supplementation with the downstream product pyruvate can enhance glycolysis and oxidative phosphorylation levels in CD8^+^ T cells (48). Research has shown that supplementing pyruvate in clear cell renal cell carcinoma enhances the activation levels of tumor-infiltrating CD8^+^ T cells (49). Additionally, a study has shown that PEP can prevent excessive accumulation of ROS and act as an antioxidant, promoting the survival of myeloid-derived suppressor cells (28). Our *in vitro* experimental results demonstrated that PEP could reduce mitochondrial ROS and pyroptosis in CD4^+^ T cells, providing a theoretical basis for its clinical translation. However, the effect of PEP on mitochondrial ROS and pyroptosis still requires further validation in animal models. The exact mechanism by which PEP affects pyroptosis remains unclear and may involve induction of ROS scavengers or enzymes capable of catalyzing redox reactions to reduce ROS production, warranting further investigation. Additionally, we also observed that PEP supplementation could significantly reduce the level of CD4^+^ T cell pyroptosis but could not completely restore it to normal level. This phenomenon may be attributed to ENO2’s regulation of pyroptosis through not only the PEP metabolic pathway but also its non-enzymatic moonlighting functions. A study has shown that ENO2 could act as a “moonlight” protein with important roles in diverse cellular processes that are not related to its function in glycolysis (50). It could also be that ENO2 has some effect on cytoplasmic ROS in addition to its effect on mitochondrial ROS. Finally, given the crucial role of ENO2 in regulating pyroptosis, elucidating its upstream regulatory pathways is of paramount importance. Previous research suggests that suppression of HIF-1α nuclear translocation by the PI3K/AKT/mTOR pathway may contribute to reduced ENO2 expression (51). Therefore, future studies aimed at identifying and characterizing key upstream regulators of ENO2 are essential to fully elucidate the molecular basis of its control in pyroptosis.

Overall, our results discovered that ENO2 regulated CD4^+^ T cell pyroptosis and explored its underlying mechanisms. These findings provide comprehensive insight into why CD4^+^ T cell numbers cannot fully recover in INR patients. The antioxidant PEP may hold the key to reducing pyroptosis levels, making pyroptosis a potential target for improving the clinical outcome of HIV infection treatment.

### Conclusions

In summary, we illustrated that the pyroptosis level is highly expressed in INRs and negatively correlated with the CD4^+^ T cell count. The CD4^+^ T cell pyroptosis in INRs preferentially occurs in more differentiated, exhausted, and activated cell subsets. ENO2 is lowly expressed in INRs and is closely related to cell pyroptosis, functionally, which regulates the pyroptosis of CD4^+^ T cells in HIV patients through mitochondrial ROS. Notably, exogenous PEP supplementation helps to restore this phenomenon. Taken together, our findings clarify the role of ENO2 in regulating CD4^+^ T cell pyroptosis in INRs and explore its potential mechanism, which may provide new targets for improving immune reconstitution and intervention in HIV infection.

## Data Availability

The data sets generated or analyzed during the current study are available in GEO DataSets (https://www.ncbi.nlm.nih.gov/gds). All data generated or analyzed during this study are available from the corresponding author on reasonable request and are included in this published article (and its supplemental material).

## SUPPLEMENTAL MATERIAL

Figure S1 mbio.01702-25-s0001.tiff The pyroptosis of CD8^+^ T cells is increased in patients with INR and is associated with disease progression.

Figure S2 mbio.01702-25-s0002.tif INR patients exhibited elevated levels of IL-1β release from CD4^+^ T cells, as well as increased mRNA expression of IL-18 and GSDMD and the statistical analysis of the proportion of pyroptosis in each CD4^+^ T cell subset between the INR and IR groups.

Figure S3 mbio.01702-25-s0003.tif Elevated levels of the apoptosis marker caspase-3 in CD4^+^ T cells of INR, and PEP attenuates the effect of ENO2 on apoptosis.

Figure S4 mbio.01702-25-s0004.tiff Downregulated differentially expressed genes in PD-1^+/−^ CD4^+^ T cells are predominantly enriched in biosynthesis of amino acid, glycolysis/gluconeogenesis, and HIF-1 signaling pathways.

Figure S5 mbio.01702-25-s0005.tif The percentage of CD4^+^ T cell death after the knockdown of ENO2 and the knockdown efficiency of ENO2 was verified by RT-qPCR.

Figure S6 mbio.01702-25-s0006.tiff ENO2^low^ and ENO2^high^ CD4^+^ T cells differentially expressed genes based on GSE18233.

Figure S7 mbio.01702-25-s0007.tif Detection of cytoplasmic ROS after inhibition of ENO2.

Figure S8 mbio.01702-25-s0008.tif Effects of supplementing PEP following ENO2 inhibition on caspase-1 in CD4^+^ T cells of ART-naïve HIV-infected individuals.

Figure S9 mbio.01702-25-s0009.tif Effects of supplementing PEP following ENO2 inhibition on activation, exhaustion, and differentiation phenotypes of CD4^+^ T cells.

Supplemental Material mbio.01702-25-s0010.docx Tables S1 and S2 and supplemental figure legends.

ASM does not own the copyrights to Supplemental Material that may be linked to, or accessed through, an article. The authors have granted ASM a non-exclusive, world-wide license to publish the Supplemental Material files. Please contact the corresponding author directly for reuse.
