# Supplementary figures and images for: ENO2 regulates CD4+ T cell pyroptosis via mitochondrial ROS to drive immunological non-response in HIV infection
Source: mBio. 2025 Sep 25;16(11):e01702-25. doi: 10.1128/mbio.01702-25 (PMC12607881; doi:10.1128/mbio.01702-25)

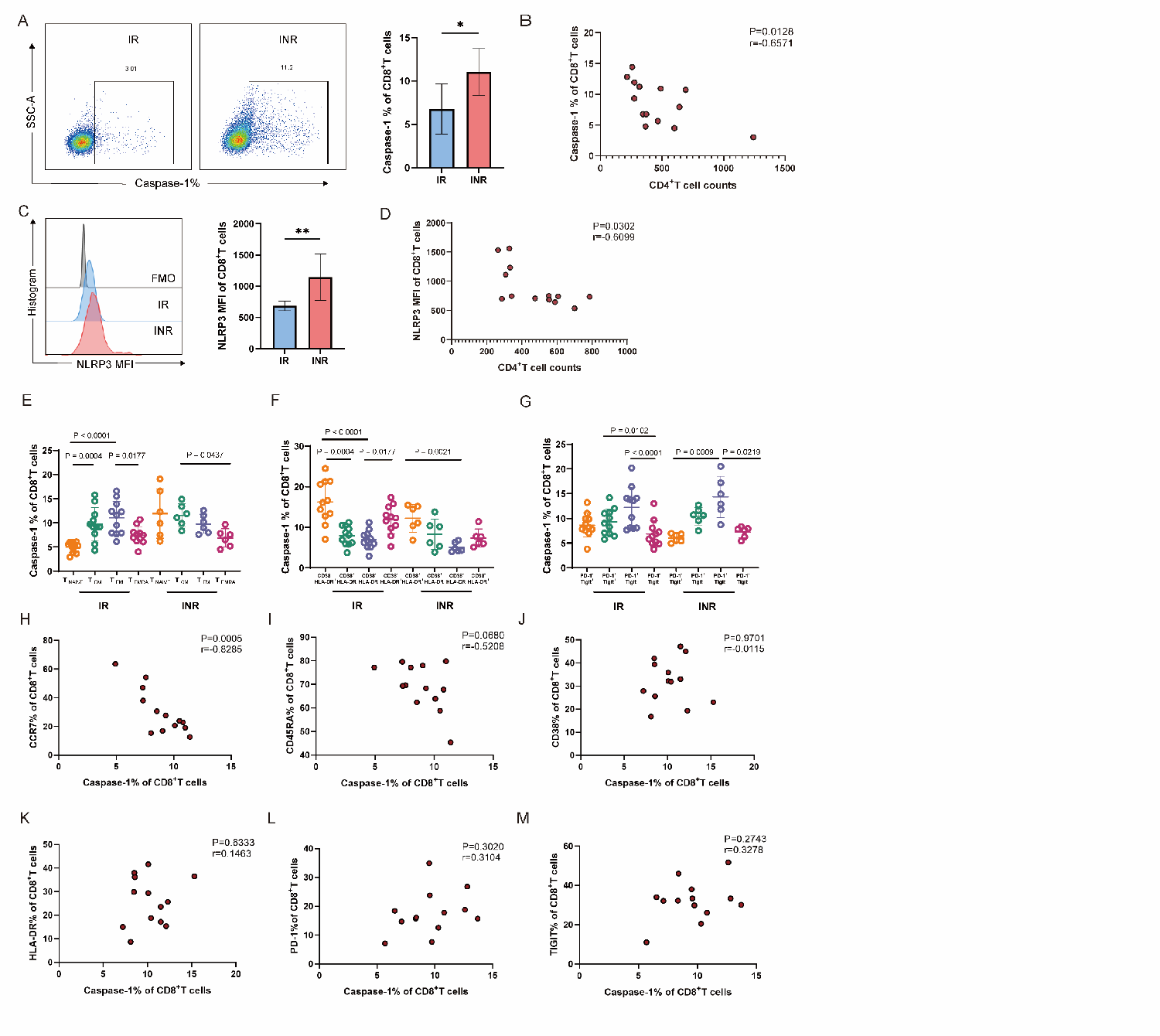

Supplement: Figure S1 — The pyroptosis of CD8+ T cells is increased in patients with INR and is associated with disease progression. [file mbio.01702-25-s0001.tiff]

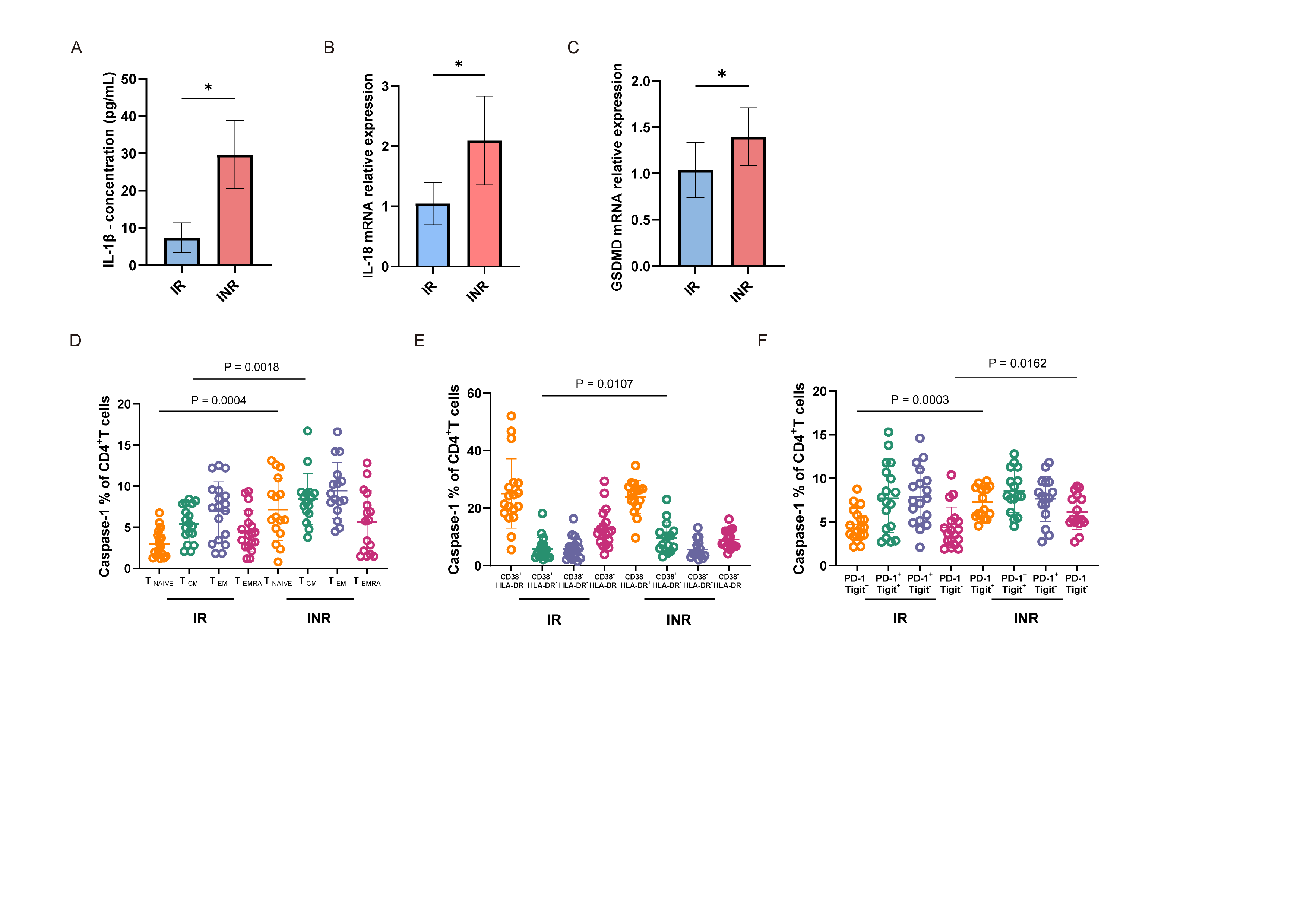

Supplement: Figure S2 — INR patients exhibited elevated levels of IL-1β release from CD4+ T cells, as well as increased mRNA expression of IL-18 and GSDMD and the statistical analysis of the proportion of pyroptosis in each CD4+ T cell subset between the INR and IR groups. [file mbio.01702-25-s0002.tif]

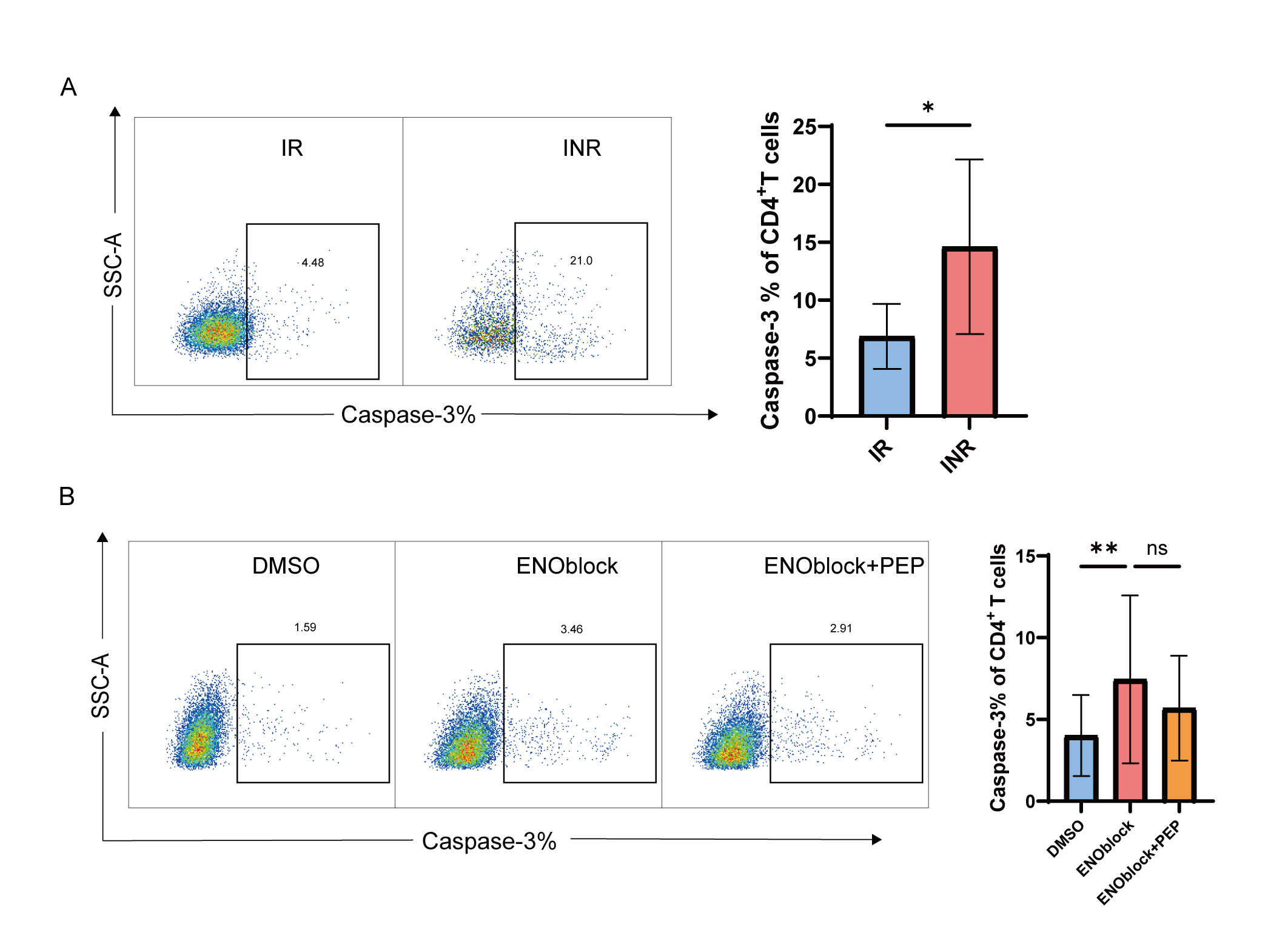

Supplement: Figure S3 — Elevated levels of the apoptosis marker caspase-3 in CD4+ T cells of INR, and PEP attenuates the effect of ENO2 on apoptosis. [file mbio.01702-25-s0003.tif]

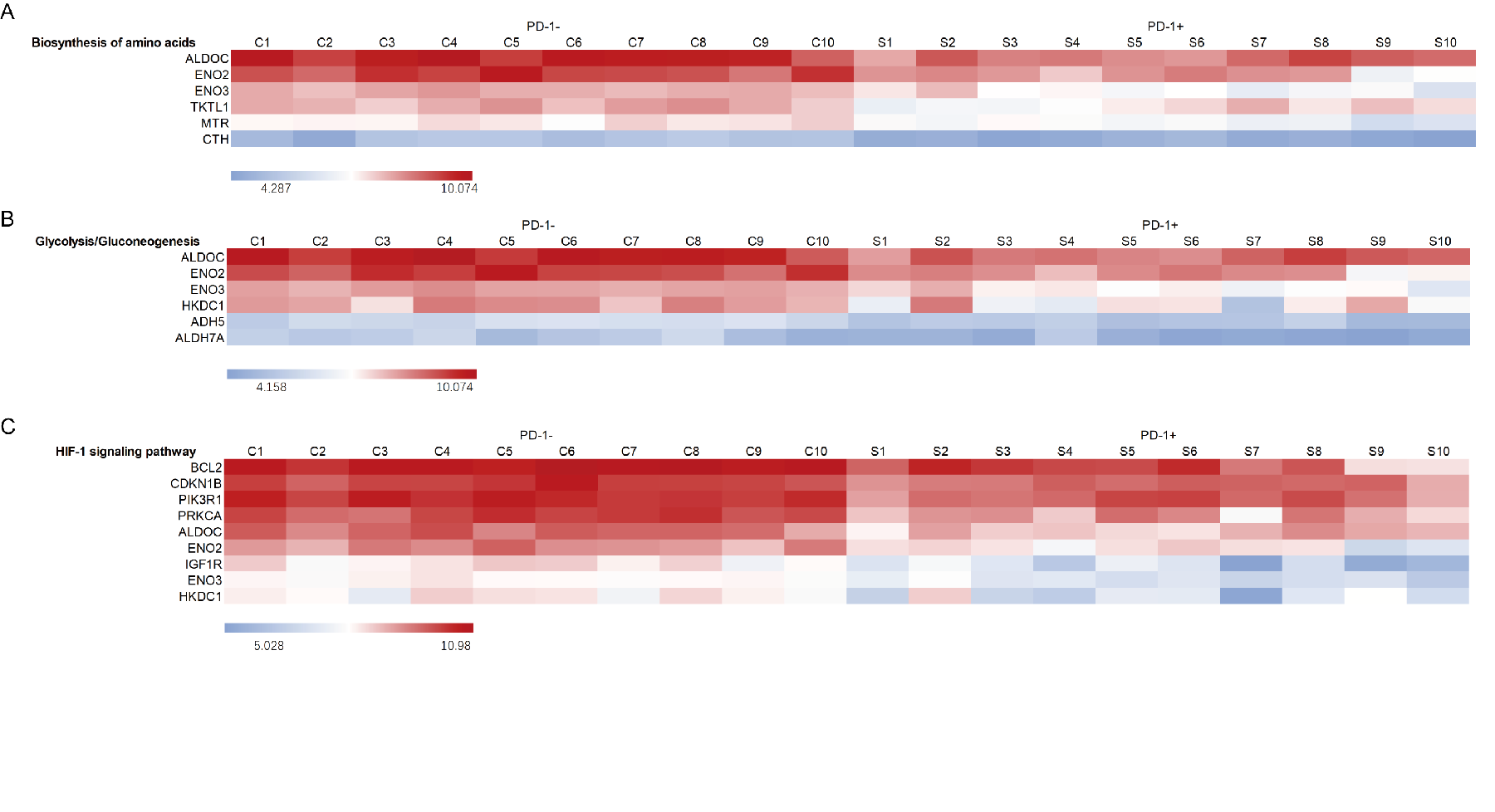

Supplement: Figure S4 — Downregulated differentially expressed genes in PD-1+/− CD4+ T cells are predominantly enriched in biosynthesis of amino acid, glycolysis/gluconeogenesis, and HIF-1 signaling pathways. [file mbio.01702-25-s0004.tiff]

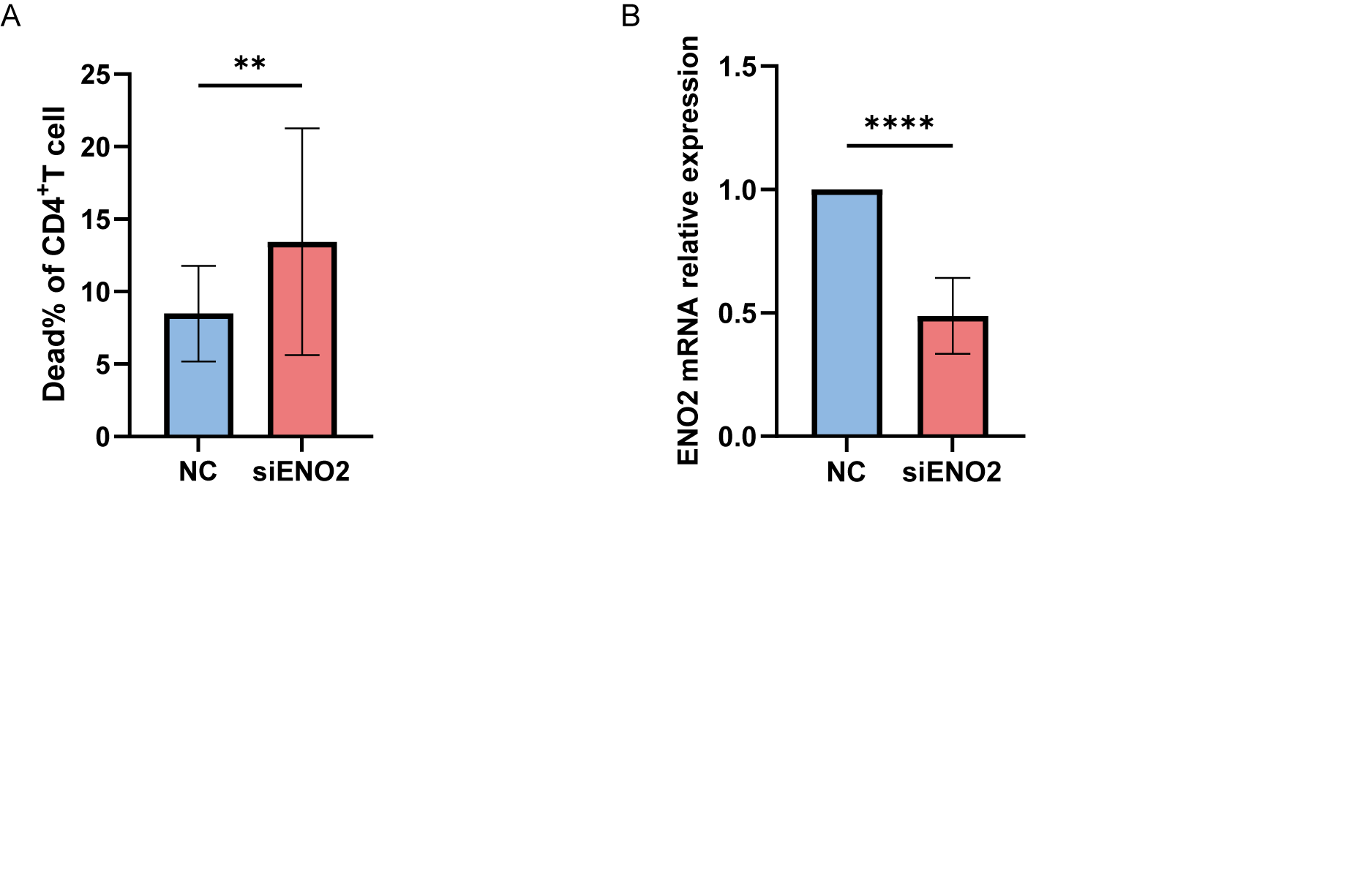

Supplement: Figure S5 — The percentage of CD4+ T cell death after the knockdown of ENO2 and the knockdown efficiency of ENO2 was verified by RT-qPCR. [file mbio.01702-25-s0005.tif]

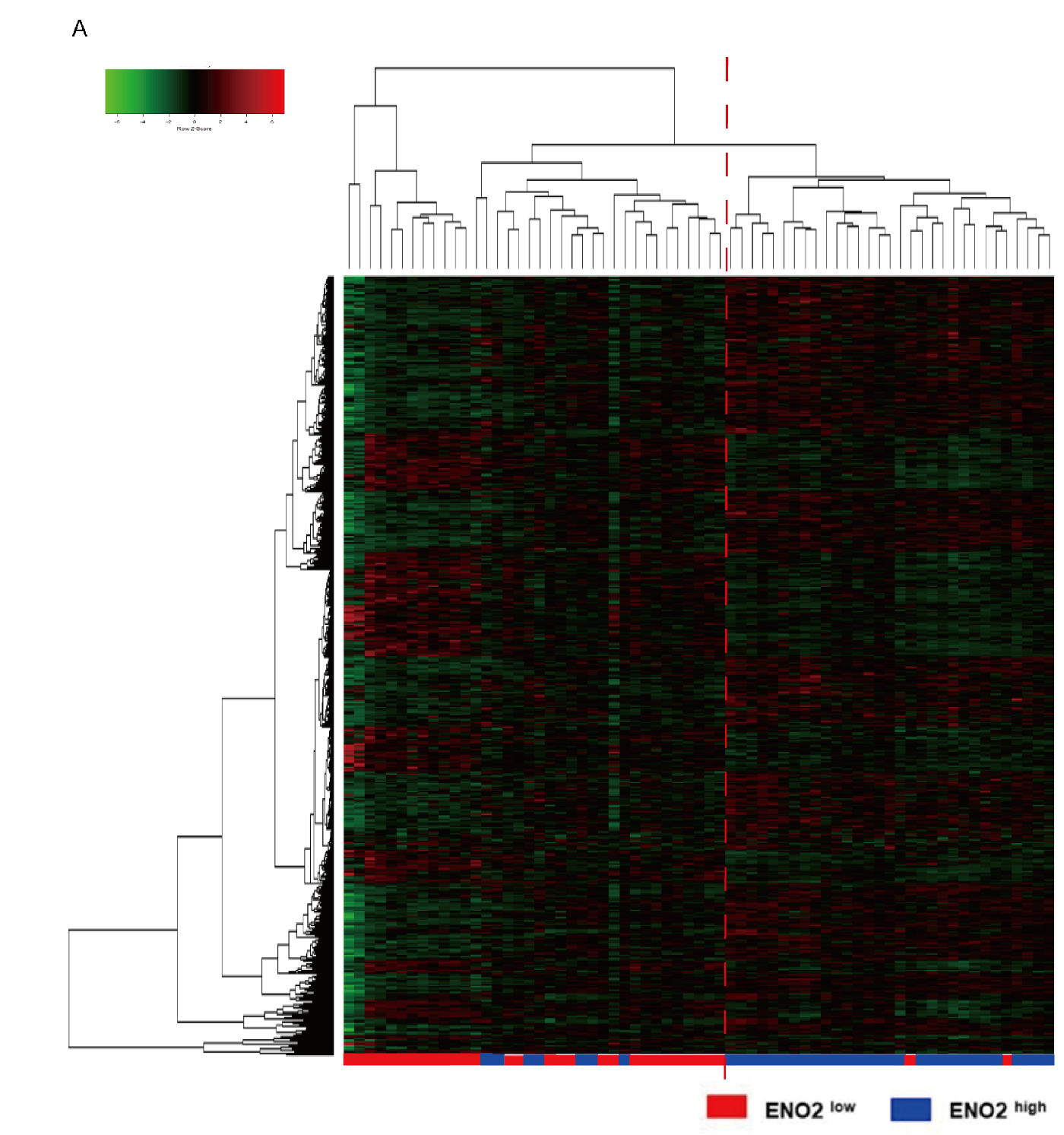

Supplement: Figure S6 — ENO2low and ENO2high CD4+ T cells differentially expressed genes based on GSE18233. [file mbio.01702-25-s0006.tiff]

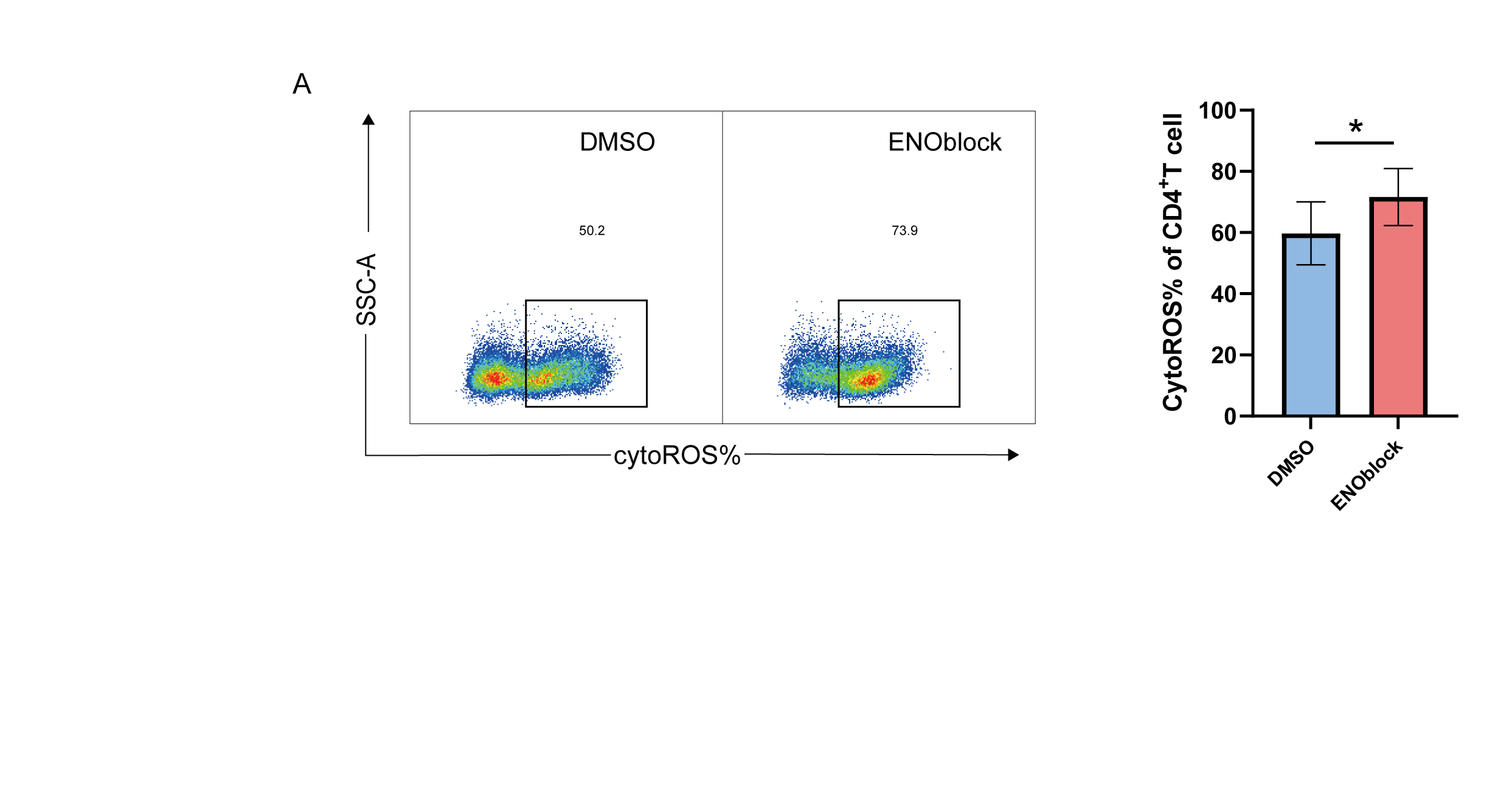

Supplement: Figure S7 — Detection of cytoplasmic ROS after inhibition of ENO2. [file mbio.01702-25-s0007.tif]

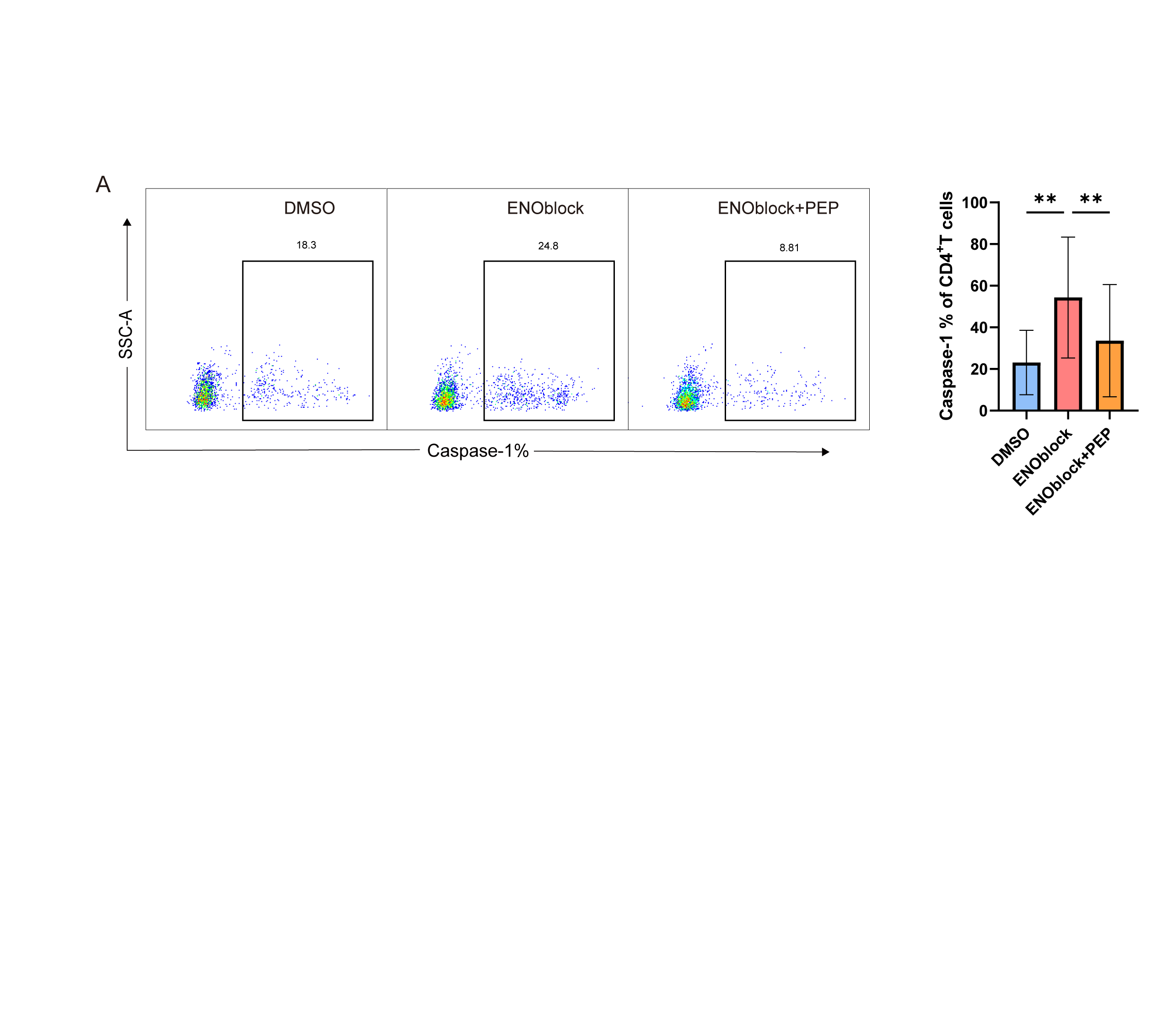

Supplement: Figure S8 — Effects of supplementing PEP following ENO2 inhibition on caspase-1 in CD4+ T cells of ART-naïve HIV-infected individuals. [file mbio.01702-25-s0008.tif]

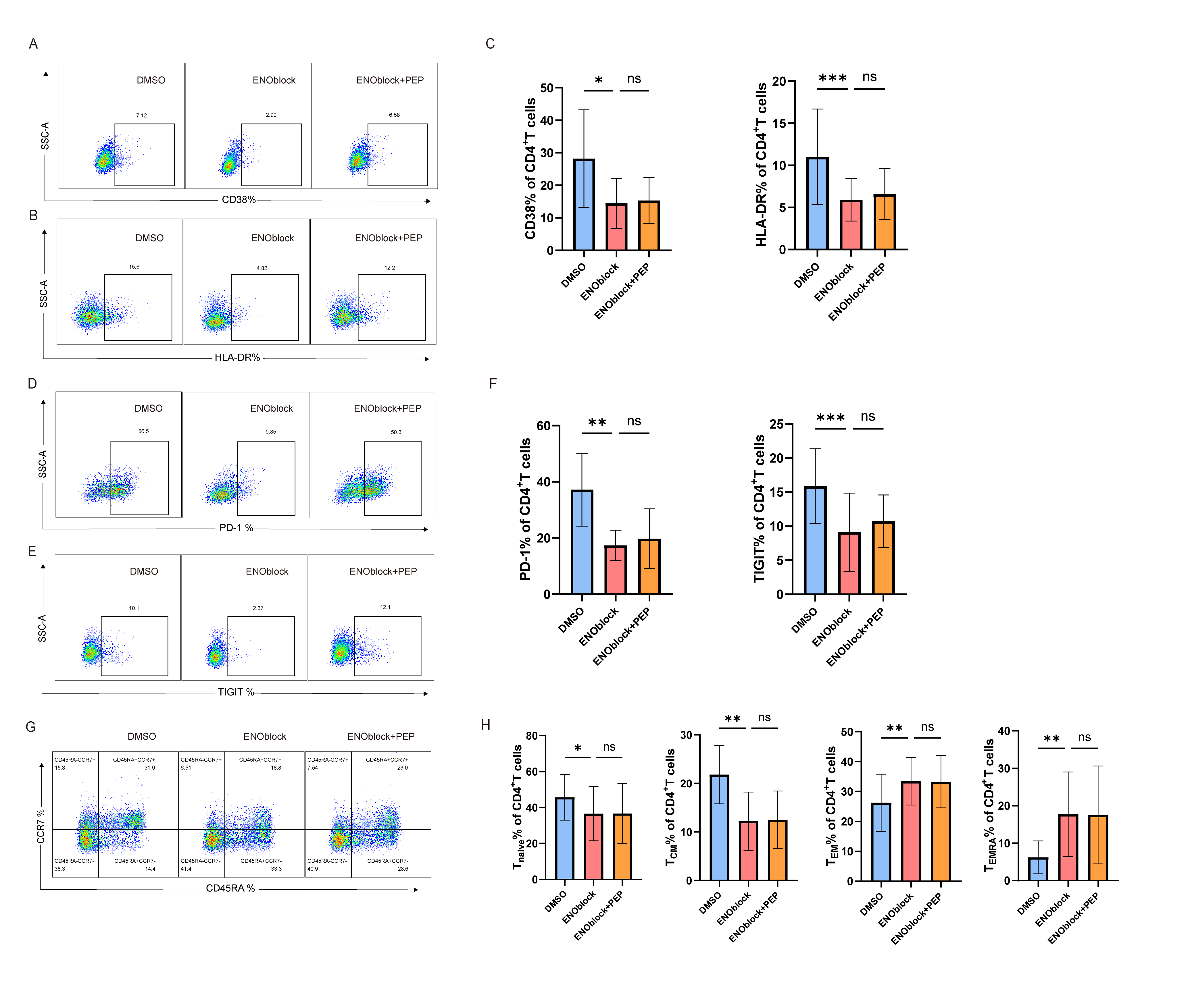

Supplement: Figure S9 — Effects of supplementing PEP following ENO2 inhibition on activation, exhaustion, and differentiation phenotypes of CD4+ T cells. [file mbio.01702-25-s0009.tif]
