# Supplementary material for: ENO2 regulates CD4+ T cell pyroptosis via mitochondrial ROS to drive immunological non-response in HIV infection
Source: mBio. 2025 Sep 25;16(11):e01702-25. doi: 10.1128/mbio.01702-25 (PMC12607881; doi:10.1128/mbio.01702-25)
Supplement: Supplemental Material — Tables S1 and S2 and supplemental figure legends. [file mbio.01702-25-s0010.docx]

**Supplementary Materials**

**Table S1.** **Basic information and clinical characteristics of subjects**

|  | IR | INR |
| --- | --- | --- |
| Cases（n） | 136 | 49 |
| Age (years, median with range) | 19-79 (35) | 22-74 (42) |
| CD4^+^ T cell counts (cells/μl, median with range) | 351-1706 (651) | 88-347 (286) |
| ART-DATE (months, median with range) | 19-162 (71) | 30-173 (67) |

**Table S2. Sequences of the primers used in this study**

| Primer | Direction | Sequences |
| --- | --- | --- |
| ENO2 | Forward  Reverse | 5’-CCGGGAACTCAGACCTCATC-3’  5’-CTCTGCACCTAGTCGCATGG-3’ |
| ENO3 | Forward  Reverse | 5’-GGCTGGTTACCCAGACAAGG-3’  5’-TCGTACTTCCCATTGCGATAGAA-3’ |
| β-actin | Forward  Reverse | 5’-CCTGGCACCCAGCACAAT-3’  5’-GGGCCGGACTCGTCATAC-3’ |

ENO2 Enolase 2, ENO3 Enolase 3

**Supplementary Figure 1: The pyroptosis of CD8^+^ T cells is increased in patients with INR and is associated with disease progression**

A. Statistical graph of the difference in the percentage of Caspase-1, an indicator of CD8^+^ T cell pyroptosis, between IR and INR patients on the right, and a typical flow graph of Caspase-1 on the left (IR: n = 8, INR: n = 6); B. Statistical graph of the correlation between the percentage of Caspase-1 expression and the CD4^+^ T cells count (IR: n = 8, INR: n = 6); C. Difference in NLRP3 inflammasome MFI of CD8^+^ T cells between IR and INR patients on the right, and typical flow graph of NLRP3 on the left (IR: n = 7, INR: n = 6); D. Correlation between NLRP3 MFI of CD8^+^ T cells and CD4^+^ T cell counts (IR: n = 7, INR: n = 6); E. Statistical graph of the proportion of CD8^+^ T cell pyroptosis indicator Caspase-1 in each differentiated subset (IR: n = 11, INR: n = 6); T _NAÏVE_: CD45RA^+^CCR7^+^ naïve T cell subset; T _CM_: CD45RA^-^CCR7^+^ central memory T cell subset; T _EM_: CD45RA^-^CCR7^-^ effector memory T cell subset; T _EMRA_: CD45RA^+^CCR7^-^ terminally differentiated effector memory T cell subset; F. Statistical graph of the proportion of CD8^+^ T cell pyroptosis indicator Caspase-1 in each activated subset in IR and INR patients (IR: n = 11, INR: n = 6); G. Statistical graph of the proportion of CD8^+^ T cell pyroptosis indicator Caspase-1 in each exhausted subset in IR and INR patients (IR: n = 11, INR: n = 6); H-M. Correlation analysis of CD8^+^ T cell pyroptosis indicator Caspase-1% with differentiated indicators CCR7 and CD45RA, activated indicators CD38 and HLA-DR, exhausted indicators of PD-1 and TIGIT, (IR: n = 13). Data are analyzed by unpaired t-test in A and C; Spearman correlation analysis in B, D; Friedman test in E-G; Pearson correlation analysis in H-M. **P < 0.01.

**Supplementary Figure 2: INR patients exhibited elevated levels of IL-1β release from CD4⁺ T cells, as well as increased mRNA expression of IL-18 and GSDMD and the statistical analysis of the proportion of pyroptosis in each CD4^+^ T cell subset between the INR and IR groups.**

A. Negatively selected ART-treated HIV-infected CD4^+^ T cells were pretreated by adding Nigericin 5 μM for 24 h. The cell secretion supernatants were collected, and the IL-1β expression level was detected by ELISA kit (IR: n = 24; INR: n = 32). B. Statistical graph of the difference in IL-18 mRNA reactive expression of CD4^+^ T cell (IR: n = 4; INR: n = 4). C. Statistical graph of the difference in GSDMD mRNA reactive expression of CD4^+^ T cell (IR: n = 9; INR: n = 9). D. Statistical graph of the proportion of CD4^+^ T cell pyroptosis indicator Caspase-1 in each differentiated subset (IR: n = 18, INR: n = 16); T _NAÏVE_: CD45RA^+^CCR7^+^ naïve T cell subset; T _CM_: CD45RA^-^CCR7^+^ central memory T cell subset; T _EM_: CD45RA^-^CCR7^-^ effector memory T cell subset; T _EMRA_: CD45RA^+^CCR7^-^ terminally differentiated effector memory T cell subset; E. Statistical graph of the proportion of CD4^+^ T cell pyroptosis indicator Caspase-1 in each activated subset in IR and INR patients (IR: n = 18, INR: n = 16); F. Statistical graph of the proportion of CD4^+^ T cell pyroptosis indicator Caspase-1 in each exhausted subset in IR and INR patients (IR: n = 18, INR: n = 16); Data are analyzed by Mann-Whitney test in A and D-F; unpaired t-test in B-C. *P < 0.05.

**Supplementary Figure 3: Elevated levels of the apoptosis marker Caspase-3 in CD4^+^ T cells of INR, and PEP attenuates the effect of ENO2 on apoptosis.**

A. Statistical graph of the difference in the percentage of Caspase-3, an indicator of CD4^+^ T cell apoptosis, between IR and INR patients on the right, and a typical flow graph of Caspase-3 on the left (IR: n = 8, INR: n = 6). B Negatively selected ART-treated HIV-infected T cells, 10 μM ENOblock was added, incubated with or without 10 μM PEP for 24 hours to detect levels of Caspase-3, typical flow charts are shown on the left, and the statistical graph of the percentage is shown on the right (n = 12). Data are analyzed by Mann-Whitney test in A; Friedman test in B. **P < 0.01, *P < 0.05.

**Supplementary Figure 4: Down-regulated differentially expressed genes in PD-1^+/-^ CD4^+^ T cells are predominantly enriched in biosynthesis of amino acid, glycolysis/gluconeogenesis and HIF-1 signaling pathways**

A. Biosynthesis of amino acid pathway: ALDOC, ENO2, ENO3, TKTL1, MTR, CTH; B. Glycolysis/gluconeogenesis pathway: ALDOC, ENO2, ENO3, HKDC1, ADH5, ALDH7A; C. HIF1 signaling pathway: BCL2, CDKN1B, PIK3R1, PRKCA, ALDOC, ENO2, IGF1R, ENO3, HKDC1.

**Supplementary Figure 5: The percentage of CD4^+^ T cell death after the knockdown of ENO2 and the knockdown efficiency of ENO2 was verified by RT-qPCR**A. Statistical graph of the difference in death percentage of CD4^+^ T cells after ENO2-knockdown (n=18). B. Statistical graph of the difference in ENO2 mRNA reactive expression of CD4^+^ T cell (n = 20). Data are analyzed by Wilcoxon signed rank test in A; Ratio paired t test in B. ****P < 0.0001, **P < 0.01.

**Supplementary Figure 6: ENO2^low^ and ENO2^high^ CD4^+^ T cells differentially expressed genes based on GSE18233**

A. Heatmap showing differentially expressed genes from ENO2^low^ and ENO2^high^ CD4^+^ T cells. Each row represents a gene and each line represents a sample. Red represents higher expression and green represents lower expression.

**Supplementary Figure 7: Detection of** **cytoplasmic ROS after inhibition of ENO2**

A. Negatively selected CD4^+^ T cells from PBMC of HIV-infected patients after ART, cytoplasmic ROS was detected 24 hours after the addition of 10 μM ENOblock, typical flow chart on the left, and percentage of cytoplasmic ROS on the right (n = 7). Data are analyzed by Wilcoxon signed rank test in A. *P < 0.05.

**Supplementary Figure 8: Effects of supplementing PEP following ENO2 inhibition on Caspase-1 in CD4^+^ T cells of ART-naïve HIV-infected individuals.**

1. Negatively selected ART-naive HIV-infected CD4^+^ T cells, after pretreatment with the antiviral drug AZT for 24 hours, 10 μM ENOblock was added, incubated with or without 10 μM PEP for 24 hours to detect levels of Caspase-1, typical flow charts are shown on the left, and the statistical graph of the percentage is shown on the right (n = 8). Data are analyzed by RM one-way ANOVA test in A. **P < 0.01.

**Supplementary Figure 9: Effects of supplementing PEP following ENO2 inhibition on activation, exhaustion, and differentiation phenotypes of CD4^+^ T cells.**

A-C. Negatively selected ART-treated HIV-infected T cells, 10 μM ENOblock was added, incubated with or without 10 μM PEP for 24 hours to detect levels of CD38 and HLA-DR, typical flow charts are shown on the left, and the statistical graph of the percentage is shown on the right (n = 12). D-F. Negatively selected ART-treated HIV-infected T cells, 10 μM ENOblock was added, incubated with or without 10 μM PEP for 24 hours to detect levels of PD-1 and TIGIT, typical flow charts are shown on the left, and the statistical graph of the percentage is shown on the right (n = 13). G-H. Negatively selected ART-treated HIV-infected T cells, 10 μM ENOblock was added, incubated with or without 10 μM PEP for 24 hours to detect levels of CD45RA and CCR7, typical flow charts are shown on the left, and the statistical graph of the percentage is shown on the right (n = 7). Data are analyzed by Friedman test in C, F and H. ***P < 0.001, **P < 0.01, *P < 0.05.
